# Endophytic *Trichoderma* and *Bacillus* isolates suppress *Lasiodiplodia theobromae*-associated dieback in blueberry under arid coastal conditions

**DOI:** 10.1038/s41598-026-46484-z

**Published:** 2026-03-31

**Authors:** William Villanueva-Olivera, Esteban Valladolid-Suyón, Mickel Palomino, Juan C. Paredes J, Johan Rivas, Richard Solórzano, María Jaramillo-Carrión

**Affiliations:** 1 Laboratorio de Fitopatología, Departamento Académico de Sanidad Vegetal, Facultad de Agronomía, Universidad Nacional Pedro Ruíz Gallo, Calle Juan XXIII 391, 14013 Lambayeque, Peru; 2 Dirección de Servicios Estratégicos Agrarios Estación Experimental Agraria Vista Florida, Instituto Nacional de Innovación Agraria (INIA), Lima, Peru; 3 Dirección de Servicios Estratégicos Agrarios, Instituto Nacional de Innovación Agraria (INIA), Centro Experimental La Molina, 15024 Lima, Peru

**Keywords:** Wood-infecting fungi, Lasiodiplodia theobromae, *Trichoderma spp*., *Bacillus spp*., Blueberry (*Vaccinium corymbosum*), Biotechnology, Microbiology, Plant sciences

## Abstract

Blueberry (*Vaccinium corymbosum* L.) production represents one of the main pillars of Peruvian agro-exports; however, its sustainability is increasingly threatened by wood-infecting fungal diseases such as dieback. This study was conducted in the Nuevo Proyecto–Olmos area (Lambayeque, Peru), covering a total cultivated area of 176 ha. The objectives were to molecularly identify the fungal pathogens associated with blueberry dieback to perform a preliminary evaluation of the antagonistic potential of endophytic microorganisms. Pathogens were isolated and characterized using morphological and molecular approaches, followed by pathogenicity tests and in vitro efficacy assays of beneficial microorganisms. Disease incidence was dominated by *Lasiodiplodia* spp., followed by *Neopestalotiopsis*, *Fusarium*, and *Diaporthe*. *Lasiodiplodia theobromae* was the most prevalent and aggressive species, confirming its association with the observed disease symptoms. For the genus *Lasiodiplodia*, temperatures between 25 and 30 °C favored mycelial growth, whereas 20 °C and 35 °C limited development. Endophytic isolates of *Trichoderma* spp. and *Bacillus* spp. inhibited pathogen mycelial growth by more than 60% under in vitro conditions. These findings highlight the potential of beneficial microorganisms as preliminary candidates for the biological control of wood-infecting fungi in blueberry production systems.

**Supplementary Information:**

The online version contains supplementary material available at 10.1038/s41598-026-46484-z.

## Introduction

Blueberry (*Vaccinium corymbosum* L.) has a high commercial demand, mainly due to its wide range of consumption forms, including fresh fruit, extracts, jams, and pharmaceutical products. This demand is largely driven by its high content of antioxidant compounds, which neutralize free radicals harmful to human health^1^. In 2024, Peru consolidated its position as the world’s leading blueberry exporter, surpassing USD 2.27 billion in export value, representing a 35% increase compared to the previous year and a total volume exceeding 326,000 tons. Organic production accounted for more than 11% of total exports^2^. This sustained growth has been supported by the expansion of productive areas in regions such as La Libertad, Lambayeque, Lima, Áncash, Ica, and Cajamarca, as well as the incorporation of new production zones including Piura and Moquegua. In parallel, varietal diversification has increased to more than 65 cultivated cultivars, with Ventura and Biloxi being the predominant ones^3,4^. In this context, the Nuevo Proyecto–Olmos area has experienced significant growth, becoming a strategic hub for foreign exchange generation and employment within the Peruvian economy^5^.

However, this rapidly expanding sector is increasingly threatened by the incidence of wood-infecting fungi that compromise both productivity and fruit quality, representing a major challenge for blueberry growers worldwide^6^. Dieback or stem blight is among the most destructive diseases affecting blueberry plantations and is predominantly associated with wood-inhabiting fungi belonging to the family *Botryosphaeriaceae*, including *Lasiodiplodia theobromae*, *Botryosphaeria dothidea*, *Neofusicoccum parvum*, and *Neopestalotiopsis* spp.^7–9^. These pathogens colonize woody tissues and may remain latent for extended periods, becoming active under environmental stress conditions, which results in tissue necrosis, reduced plant vigor, yield decline, and eventual plant death^10^.

Within this pathological complex, species of the genus *Lasiodiplodia* constitute a group of morphologically similar yet genetically diverse fungi that have been reported as causal agents of dieback and decline diseases in a wide range of woody crops, including blueberry. Accurate identification of these species is essential due to differences in aggressiveness, epidemiological behavior, and responses to management strategies, which justifies the use of molecular tools for their precise characterization.

Chemical control strategies against wood diseases are often limited in effectiveness and raise environmental and regulatory concerns. Consequently, integrated disease management approaches that incorporate the use of beneficial microorganisms are increasingly promoted as sustainable alternatives^11^. Among the most promising biocontrol agents are strains of *Trichoderma* and *Bacillus*, which are well known for their ability to suppress phytopathogens through multiple mechanisms, including the production of lytic enzymes, antifungal metabolites, and the induction of plant defense responses^12,13^. Endophytic strains of these genera offer an additional ecological advantage, as their capacity to colonize internal plant tissues enhances persistence and effectiveness against wood-infecting pathogens^14,15^.

Despite increasing interest in biological control of wood-infecting pathogens in blueberry, information on the preliminary antagonistic performance of locally obtained endophytic microorganisms under arid coastal conditions remains limited.

Within this framework, the objective of the present study was to molecularly identify the main wood-infecting fungi associated with blueberry dieback (main Lasiodiplodia isolates LS1–LS7) and to conduct a preliminary screening of endophytic *Trichoderma* spp. and *Bacillus* spp. isolates with potential antagonistic activity against these pathogens under in vitro conditions. This approach aimed to generate baseline information for the future development of biological control strategies targeting stem blight in blueberry production systems. We hypothesized that endophytic isolates of Trichoderma and Bacillus obtained from blueberry tissues would exhibit differential antagonistic activity against Lasiodiplodia species under arid coastal conditions.

## Materials and methods

### Study area

The study was conducted in commercial blueberry (*Vaccinium corymbosum* L.) plantations located in the Nuevo Proyecto–Olmos area, Lambayeque, northern Peru. Field evaluations were carried out in three commercial farms: Pesquera (6°04′25″ S, 79°56′57″ W; 90 ha), B11 (6°06′17″ S, 79°57′22″ W; 50 ha), and Compostera (6°05′43″ S, 79°57′18″ W; 36 ha) (Supplementary Material S1). At each site, stem samples exhibiting visible disease symptoms as well as asymptomatic plant material were collected. In addition, associated phytopathogenic fungi and beneficial endophytic microorganisms were isolated from the sampled tissues. All isolation, culturing, and characterization procedures were performed at the Phytopathology Laboratory of the Faculty of Agronomy, Universidad Nacional Pedro Ruiz Gallo, and at the Microbiology Laboratory of the Research and Development (R&D) Department of Ingleby Farms.

### Sampling of blueberry plant tissues

A total of 54 blueberry plant tissue samples were collected using a probabilistic sampling approach, assuming an estimated disease prevalence of 90%, based on Hernández et al.^16^ and the results of a preliminary pilot survey. For pathogenicity tests, 140 two-year-old blueberry plants were used and inoculated with the previously isolated wood-infecting fungi. Each fungal isolate was inoculated on ten plants, and a total of fifteen stems per isolate were selected as subsampling units for lesion development assessment. In plants where more than one stem was inoculated, lesion length measurements were averaged at the plant level prior to statistical analysis to avoid pseudoreplication. In the in vitro antagonism assay, four beneficial microorganisms isolated from healthy plants within the same production system were evaluated. These isolates were selected based on their biocontrol potential to assess their inhibitory effects against the identified wood-infecting pathogens.

### Assessment of dieback incidence in blueberry crop

The incidence of blueberry dieback was assessed in the Pesquera, B11, and Compostera farms, covering a total cultivated area of 176 ha. Plant evaluations and sample collection were conducted following a zigzag sampling pattern, assessing approximately 1.5% of the total plants at each site. During field surveys, plants were inspected for characteristic symptoms of dieback. Disease incidence was calculated as the proportion of symptomatic plants relative to the total number of evaluated plants. A plant was considered diseased when at least one symptom or sign associated with dieback was observed.

### Isolation of the causal agent of blueberry dieback

Stem samples affected by dieback symptoms collected in the field were placed in coded sterile bags and transported to the Phytopathology Laboratory of the Universidad Nacional Pedro Ruiz Gallo. Tissue fragments taken from the disease advancement margin were surface-disinfected with 1% sodium hypochlorite, rinsed with sterile distilled water, and plated onto potato dextrose agar (PDA). Plates were incubated at 28 ± 1 °C for 7 days, with three replicates per sample. Developed colonies were selected, purified, and grouped based on their macroscopic and microscopic characteristics using the taxonomic keys of Barnett & Hunter,^17^. Monoconidial cultures were obtained from each isolate following the dilution procedures described by Alama et al.^18^ and Rodríguez-Gálvez et al.^7^. Pure isolates were maintained on PDA slants and stored at 5 °C for further analyses.

### Pathogenicity tests on blueberry plants

Monoconidial cultures were inoculated onto healthy blueberry plants by placing agar plugs containing actively growing mycelium onto apical stem wounds, while non-colonized PDA agar discs were used as controls. Inoculation was performed using 5-mm-diameter mycelial discs obtained from actively growing cultures of each pathogen isolate. Although the first formal disease assessment was conducted at 7 days after inoculation, the earliest symptoms were observed at 3 days after inoculation with isolate LS5, appearing as localized tissue darkening around the inoculation point that progressively developed into typical dieback symptoms. At 40 days after inoculation, longitudinal stem sections revealed internal necrosis of vascular bundles characteristic of dieback. Microscopic examination of necrotic tissues further confirmed pathogen colonization through the observation of pycnidia.

Plants were maintained under non-controlled field conditions for 40 days, during which climatic variables were recorded throughout the evaluation period (Supplementary Material S2). After symptom development, the inoculated fungi were re-isolated from affected tissues through surface disinfection and incubation on PDA. The recovered colonies were subsequently compared with the original isolates based on their macroscopic and microscopic characteristics to confirm pathogenicity.

### Morphological characterization of the etiological agent of dieback

From cultures that developed characteristic disease symptoms, colony morphology and growth rate were evaluated on potato dextrose agar (PDA) and cornmeal agar (CMA) at different temperatures (20, 25, 30, and 35 °C). Daily radial growth was determined using agar plugs (5 mm diameter) containing actively growing mycelium placed at the center of each Petri dish, with four replicates per isolate and temperature. In addition, microscopic characteristics of fungal structures were described, including spore size, shape, and pigmentation. Conidial morphology was further characterized for each genus. Lasiodiplodia isolates initially produced hyaline conidia that later became dark brown, ellipsoidal in shape, measuring 20.40–30.27 × 10.12–15.24 µm. Neopestalotiopsis isolates exhibited predominantly hyaline, multicellular fusiform conidia with characteristic apical appendages, measuring 20.27–25.98 × 6.47–8.45 µm. Fusarium isolates produced hyaline, falcate, multicellular macroconidia with approximate dimensions of 25.74–45 × 3–5 µm. Diaporthe isolates formed hyaline conidia ranging from ellipsoidal to fusiform in shape, measuring 6–10 × 2–3 µm. Species-level identification was performed based on morphological criteria, following the descriptions of Sutton^19^ and the taxonomic keys of the Commonwealth Mycological Institute (CMI), United Kingdom.

### Molecular identification of wood-infecting fungi

Fungal isolates were cultured on potato dextrose agar (PDA) at 28 °C for seven days, and genomic DNA was extracted from mycelial tissue using an extraction buffer and proteinase K protocol, followed by purification with chloroform and phenol–chloroform–isoamyl alcohol. DNA was precipitated with isopropanol and ethanol and subsequently resuspended in Tris–EDTA buffer. PCR amplification was performed using the universal primers ITS1 and ITS4, and the resulting amplicons were purified and sequenced using the Sanger method. The obtained sequences were identified by BLAST searches against the NCBI database and deposited in GenBank under accession numbers MW299392, PP439605, PQ114133, UDB076775, PP859451, PQ126990, OQ316613, ON454616, FJ612924, and UDB07. Additionally, the most aggressive isolates (L05 and L07) were subjected to complementary molecular analyses through sequencing of the ITS, elongation factor 1-alpha (EF1-α), and beta-tubulin (BT2) genes. The resulting sequences were edited and analyzed using BLASTn (NCBI), and molecular identification was based on sequence similarity with reference sequences available in GenBank (PQ479076.1, MN461169.1, ON373967.1, and XM_035519539.1).

### In vitro efficacy of antagonistic endophytic microorganisms against the causal agent of dieback

#### Isolation of microorganisms

The beneficial microorganisms used in the antagonism assays were isolated from healthy blueberry tissues. Endophytic fungi were isolated following the methodology described by Alama et al.^18^. For the isolation of endophytic bacteria, plant tissues were washed, sectioned, and subjected to surface disinfection using 70% ethanol and 0.53% sodium hypochlorite, followed by rinsing with sterile distilled water. Subsequently, aliquots from the final rinse were plated onto tryptic soy agar (TSA) using the quadrant streaking technique and incubated at 28 ± 1 °C for 48 h^20^. The resulting colonies were characterized based on macroscopic and microscopic criteria, according to Barnett & Hunter^17^ and Bergey et al.^21^.

#### Antagonism assay against the causal agent of dieback during 3, 4 and 7 DAI.

**Fig. 1:**
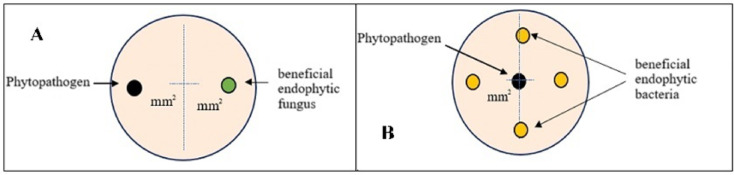
Schematic representation of the dual confrontation assays between phytopathogenic fungi and beneficial endophytic microorganisms. (**A**) Fungus–fungus interaction, in which mycelial plugs of the phytopathogen and *Trichoderma* spp. were placed at opposite sides of the Petri dish. (**B**) Fungus–bacterium interaction, where the phytopathogenic fungus was inoculated as a mycelial plug at the center of the plate, while *Bacillus* spp. were applied as bacterial inoculation points equidistant from the fungal colony to evaluate growth inhibition.

The antagonistic capacity of endophytic microorganisms was evaluated using dual confrontation assays against three highly pathogenic wood-infecting fungal isolates: LS5, LS2 (*Lasiodiplodia citricola*), and LS7 (*L. theobromae*). First six beneficial endophytic microorganisms (three *Trichoderma* spp. isolates and three *Bacillus* spp. isolates) were tested (during 3 and 7 days) on potato dextrose agar (PDA) by placing 5-mm agar plugs at opposite edges of Petri dishes (Fig. 1), with five replicates per treatment, following the methodology described by Dennis and Webster^22^. For the fungus–bacterium interaction assay, the phytopathogenic fungus was inoculated at the center of the Petri dish as a 5-mm mycelial plug, while *Bacillus* spp. were applied as 3–4 equidistant bacterial inoculation spots around the fungal colony. Following this preliminary screening stage, isolates showing the highest antagonistic performance were selected for subsequent inhibition assays conducted under a poisoned medium experimental framework. Subsequently, a confirmatory inhibition assay was performed during 4 and 7 DAI, with two isolated fungal (T1 as Tr06 and T2 as Tr01) and three bacterial treatments (BV01, T4 and T5).

Mycelial growth was quantified using ImageJ software (version 1.54), and the percentage of mycelial growth inhibition was calculated relative to the control. For *Trichoderma* spp. treatments, antagonistic efficacy was additionally assessed using the parasitism scale proposed by Bell et al.^23^ to compare interaction outcomes among treatments (Supplementary Material S3).

### Propagation and in vitro efficacy of beneficial microorganisms

#### Preparation of bacterial inoculum

Selected *Bacillus* isolates were multiplied using a culture medium composed of molasses (15 g L^−1^), yeast extract (2 g L^−1^), magnesium sulfate (7 g L^−1^), and an antifoaming agent (0.01 mL L^−1^), maintained at 28 °C. The inoculum was added at a concentration of 0.4 mL L^−1^, and microbial growth was sustained for 24 h. The resulting biomass was subsequently used in in vitro efficacy assays.

#### Preparation of fungal inoculum

Beneficial fungal isolates were multiplied using rice (900 g) as a solid substrate. The substrate was sterilized at 121 °C for 15 min and aseptically inoculated with 1 mL of a conidial suspension (1 × 10^8^ conidia mL^−1^). The inoculated bags were incubated at 30 ± 1 °C until sporulation was achieved, after which the fungal biomass was washed with sterile distilled water to obtain the final biological product.

#### Monitoring of bacterial concentration

During the multiplication process, colony-forming units (CFU) were quantified using standard microbiological techniques. Both biological products were adjusted to a final concentration of 1 × 10^9^ CFU mL^−1^ and subsequently used in the assays following the poisoned medium technique. In the case of bacterial isolates, the target concentration was naturally achieved during the fermentation process.

#### In vitro efficacy assay of biological products

The efficacy of the biological products as inhibitors of mycelial growth of the causal agents of dieback or stem blight was evaluated using the poisoned medium technique^24^. Mycelial plugs (5 mm diameter) of the phytopathogens were placed at the center of Petri dishes containing potato dextrose agar (PDA) supplemented with the biological products, including an untreated control. Plates were incubated at 25 ± 1 °C, and mycelial growth was measured daily for seven days using ImageJ software (version 1.54), with four replicates per treatment. In addition, commercial products based on *Trichoderma asperellum* (T-34®) and *Bacillus subtilis* (Bio-Splent 70WP®) were included as reference standards to compare biocontrol efficacy. The percentage of inhibition of mycelial growth (PIMG) was calculated according to the methods described by Alburqueque and Gusqui^25^ and Skidmore and Dickinson^26^ (Table 1).

$$PICM=\frac{RGC-RGT}{RGC}X 100$$

where: PICM: Percentage of inhibition of mycelial growth; RGC: Radial growth of the control colony (area); RGT: Radial growth of the treated colony (area).

**Table 1:** Treatments and doses used to evaluate the in vitro efficacy of beneficial microorganisms using the poisoned medium technique.

Treatment	Active ingredient	Dose per 100 mL	Final concentration
T1	Beneficial fungus 1(Tr06)	0.02 mL	1 × 10^9^ CFU mL^−1^
T2	Beneficial fungus 2(Tr01)	0.02 mL	1 × 10^9^ CFU mL^−1^
T3	Beneficial bacterium (Bv01)	0.1 mL	1 × 10^9^ CFU mL^−1^
T4	T-34 ® *(T. asperellum)*	0.001 gr	1 × 10^12^ CFU kg^−1^
T5	Bio-Splent 70WP*® (B. subtilis)*	0.1 mL	1 × 10^9^ CFU mL^−1^
T6	*Control* (untreated*)*	–	–

#### Statistical analysis

Pathogenicity tests were analyzed using a randomized complete block design (RCBD), whereas the dual confrontation assays and mycelial growth inhibition experiments followed a completely randomized design (CRD). Data that did not meet the assumptions of normality or homogeneity of variances were transformed using square-root or arcsine transformations to satisfy the requirements for analysis of variance (ANOVA). Statistical analyses and graphical representations were performed using Microsoft Excel 365 and RStudio (version 4.4.2). Mean comparisons were conducted using Tukey’s honestly significant difference (HSD) test at a significance level of α = 0.05, using InfoStat statistical software (version 2020e). Blocks corresponded to spatial plant position within the experimental field. Disease progress over time was additionally quantified by calculating the area under the disease progress curve (AUDPC) based on lesion length measurements recorded at each evaluation date.

All laboratory experiments were conducted under controlled laboratory conditions using standardized inoculation procedures, defined incubation regimes, and appropriate replication structures within a completely randomized experimental framework, whereas pathogenicity tests were performed under semi-field conditions.

## Results

### Assessment of dieback incidence in the blueberry crop

Characteristic disease symptoms were observed, including necrosis of stems, leaves, and shoots, which in severe cases led to defoliation and plant death. Dieback incidence reached 8% in the Pesquera farm (fifth production cycle), 11% in B11 (seventh production cycle), and 3% in Compostera (third production cycle). Across the total evaluated area (176 ha), the average incidence of the wood-infecting fungal complex was 7%, corresponding to approximately 61,600 affected plants. Cultivar-based analysis revealed that ‘Emerald’ and ‘Snowchaser’ exhibited the highest incidence levels (12% and 11%, respectively), suggesting a higher susceptibility to fungi associated with blueberry dieback (Supplementary Material S4).

### Isolation of the causal agent of dieback in the blueberry crop

**Fig. 2:**
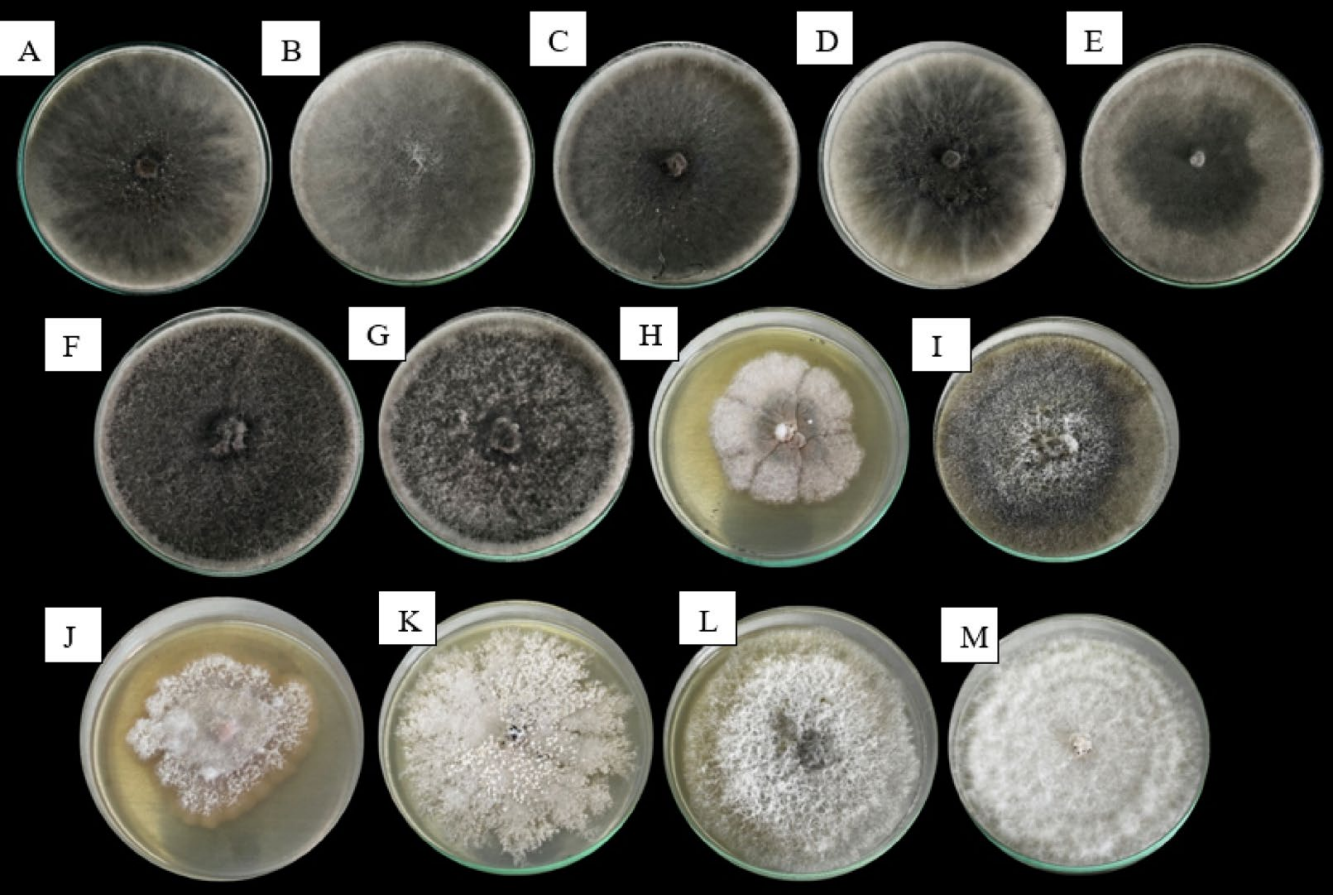
Distinct morphological features of the identified pathogens. *Note:* (**A**) *Lasiodiplodia sp.* LS1, (**B**) *Lasiodiplodia sp.* LS2, (**C**) *Lasiodiplodia sp.* LS3, (**D**) *Lasiodiplodia sp*. LS4, (**E**) *Lasiodiplodia sp*. LS5, (**F**) *Lasiodiplodia sp.* LS6, (**G**) *Lasiodiplodia sp* LS7, (**H**) *Neopestaliotopsis sp*. C1, (**I**) *Diaporthe sp.* C2, (**J**) *Fusarium sp.* C3, (**K**) *Neopestalotiopsis sp.* P1, (**L**) *Neopestalotiopsis sp.* P2 y (**M**) *Neopestalotiopsis sp*. P3.

From the 54 field samples exhibiting disease symptoms, wood-infecting fungi were isolated in 100% of the cases. *Lasiodiplodia* spp. was the predominant genus, detected in 67% of the samples, followed by *Neopestalotiopsis* spp. (19%), *Fusarium* spp. (10%), and *Diaporthe* spp. (4%). A total of 79 fungal isolates were obtained, of which 53 corresponded to *Lasiodiplodia*, 15 to *Neopestalotiopsis*, 8 to *Fusarium,* and 3 to *Diaporthe*. In addition, distinct colonial morphologies were observed within each genus (Fig. 2); therefore, each morphotype was assigned a specific code to facilitate subsequent molecular identification and downstream analyses.

### Pathogenicity tests on blueberry plants

**Fig. 3:**
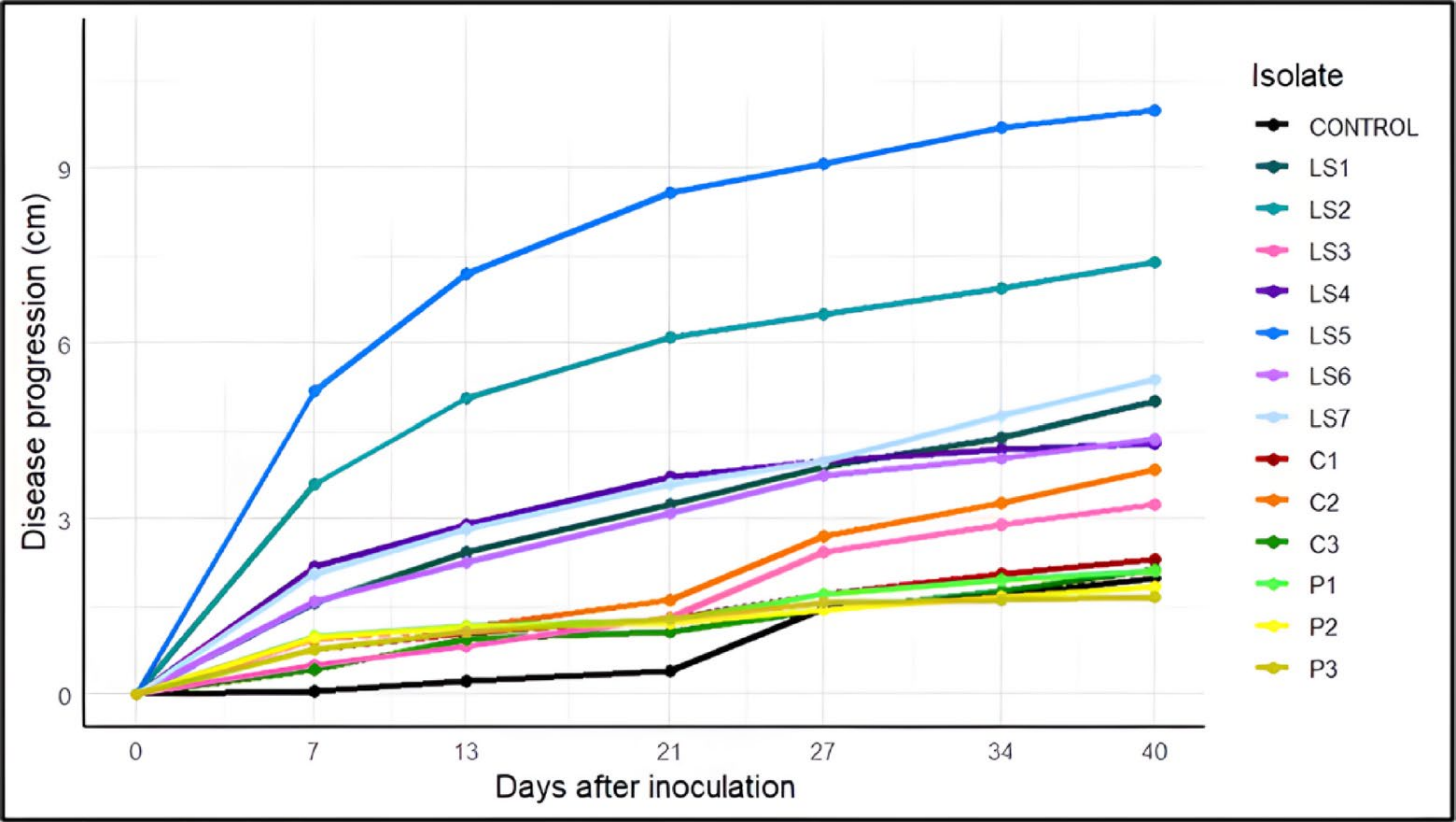
Progression of longitudinal disease development following inoculation with different wood-infecting fungal isolates. *Note:* LS1 *Lasiodiplodia sp.*, LS2 *Lasiodiplodia sp*, LS3) *Lasiodiplodia sp.*, LS4 *Lasiodiplodia sp*., LS5 *Lasiodiplodia sp*., LS6 *Lasiodiplodia sp.*, LS7 *Lasiodiplodia sp*, C1 *Neopestaliotopsis sp*., C2 *Diaporthe sp.*, C3 *Fusarium sp.*, P1 *Neopestalotiopsis sp.*, P2 *Neopestalotiopsis sp.* y P3 *Neopestalotiopsis sp*.

Pathogenicity tests conducted on blueberry plants revealed significant differences in disease progression (measured in centimeters) among the evaluated fungal isolates over time, expressed as days after inoculation (DAI). (Fig. 3; Supplementary Material S5). In addition, disease symptoms progressed over time after inoculation, with initial localized necrosis observed at 14 days, followed by increased lesion expansion and shoot dieback at 21 and 28 days, and severe vascular discoloration and tissue collapse at 40 days after inoculation (Supplementary Material S2).

Isolate LS5 exhibited the highest disease aggressiveness, followed by LS2, LS7, and LS1 (*p* < 0.05) under the experimental conditions, as evidenced by the progressive development of necrosis in the inoculated tissues. In contrast, isolates C1, C2, C3, and P3 showed limited disease progression, suggesting weak or negligible pathogenic capacity in the evaluated plant system.

Significant differences among fungal isolates were also confirmed by the analysis of the area under the disease progress curve (AUDPC). Isolate LS5 showed the highest AUDPC values, indicating greater epidemiological aggressiveness and faster lesion expansion compared with the remaining isolates. Intermediate AUDPC values were observed for LS2 and LS4, whereas isolates such as C3, P3 and the non-inoculated control exhibited the lowest disease progress over time (Table 2).

**Table 2:** Analysis of variance and mean comparison (Tukey test) for AUDPC values recorded during pathogenicity assays*: Analysis of Variance (ANOVA) for AUDPC.*

Source	Df	Sum Sq	Mean Sq	F value	*p*-value
Pathogen	13	679,310.1	52,254.621	36.9075	0.00000000
Residuals	196	277,502.0	1,415.827		

*Tukey HSD test for AUDPC*		
Treatment	AUDPC_Mean	Significance
LS5	230.66	a
LS2	163.38	b
LS4	98.54	c
LS7	98.04	c
LS1	87.84	cd
LS6	83.66	cde
C2	52.74	cdef
LS3	42.63	def
P1	40.64	ef
C1	38.64	ef
P2	37.26	ef
P3	36.70	f
C3	31.40	f
CONTROL	19.38	f

### Temperature-dependent mycelial growth and phenotypic responses of Lasiodiplodia isolates

**Fig. 4:**
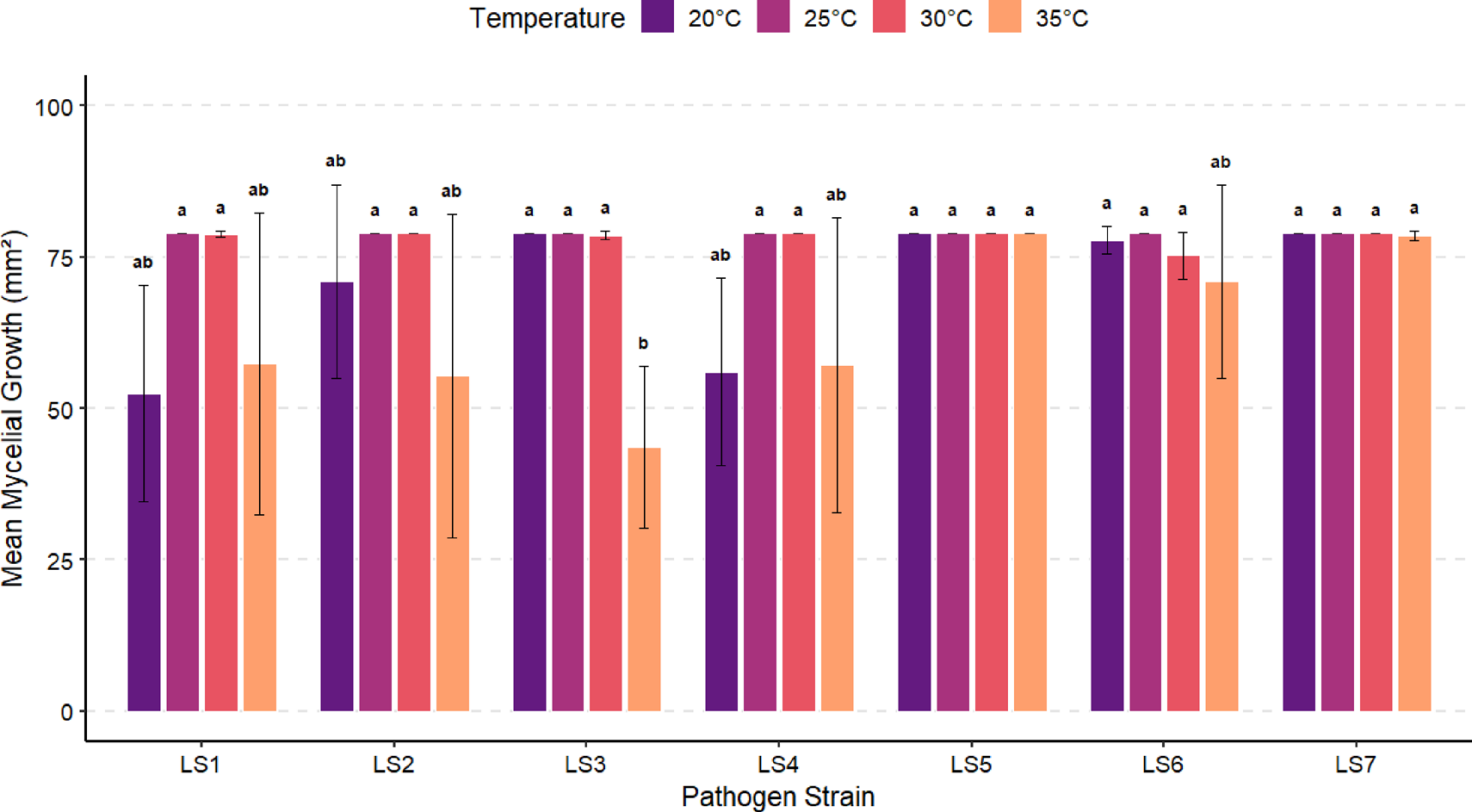
Mean mycelial growth of *Lasiodiplodia* on PDA at four temperature regimes 14 days after inoculation.

Analysis of mycelial growth of the seven *Lasiodiplodia* isolates (LS1–LS7) on PDA and CMA media under four temperature regimes (20, 25, 30, and 35 °C) revealed a pronounced effect of both substrate and temperature on daily radial expansion rates as well as on colony morphological expression (Fig. 4).

Mycelial growth of the seven isolates (LS1–LS7) showed a strong temperature dependence. At 20 °C, growth was the slowest and most heterogeneous among isolates. At 25 °C, the fastest, most uniform, and most stable growth was recorded, with most isolates covering the Petri dishes within 4–6 days; under these conditions, LS5 and LS7 consistently exhibited the highest growth rates. At 30 °C, a pattern similar to that observed at 25 °C was recorded, although with greater variability among replicates, and LS7 remained the fastest-growing isolate. In contrast, exposure to 35 °C resulted in pronounced thermal stress, markedly reducing mycelial expansion rates and prolonging plate coverage times (7–14 days or, in some cases, incomplete colonization). Overall, the favorable temperature range for mycelial growth on PDA was between 25 and 30 °C, which promoted rapid and homogeneous expansion, whereas 20 °C and particularly 35 °C significantly limited growth, highlighting the direct influence of thermal regime on fungal physiology. On CMA medium, mycelial growth was faster and more homogeneous than on PDA. At 20 °C, all isolates achieved complete plate colonization by day 6 (13.2 mm^2^ day^−1^), with LS6 standing out for early sporulation and pigmentation, while LS2 and LS4 displayed a more gradual but sustained growth pattern. Between 25 and 30 °C, mycelial expansion was uniform and rapid, reaching full coverage by day 3 (26.3 mm^2^ day^−1^), confirming that cornmeal-based medium strongly enhances mycelial growth compared with PDA under equivalent temperature conditions. At 35 °C, all isolates also achieved complete colonization within 3–4 days (19.8–26.3 mm^2^ day^−1^), maintaining high growth rates despite elevated temperature. Nevertheless, noticeable morphological changes were observed, including reddish pigmentation in isolates LS2 and LS3, indicating thermal stress responses without a substantial reduction in colonization capacity.

### Molecular identification of wood-infecting fungal isolates

To determine the taxonomic identity of the obtained isolates, the internal transcribed spacer (ITS) region was sequenced and subsequently subjected to comparative analysis using BLASTN version 2.15.0+ in combination with the UNITE specialized database (Table 3; Supplementary Material S6).

**Table 3:** Taxonomic assignment and molecular identification of the collected pathogenic species.

Code	Sample	Identified species	Sequence length (pb)	Identity (%)	Reference
PQ809537	LS01	*Lasiodiplodia sp.*	331	96.97	MW299392
PQ809538	LS02	*Lasiodiplodia citricola*	246	99.60	PP439605
PQ809539	LS03	*Lasiodiplodia iraniensis*	460	100.00	PQ114133
PQ809540	LS04	*Lasiodiplodia theobromae*	259	99.62	UDB0767750
PQ809541	LS05	*Lasiodiplodia sp.*	199	99.50	MW299486
PQ809542	LS06	*Lasiodiplodia theobromae*	207	100.00	UDB0767750
	LS07	No significant similarity	–	–	–
PQ809543	P1	*Neopestalotiopsis clavispora*	333	100.00	PP859451
PQ809544	P2	*Neopestalotiopsis sp.*	392	100.00	PQ126990
PQ809545	P3	*Neopestalotiopsis vaccinii*	561	99.64	OQ316613
PQ809534	C1	*Neopestalotiopsis sp.*	565	99.65	ON454616
PQ809535	C2	*Diaporthe sp.*	428	99.77	FJ612924
PQ809536	C3	*Fusarium solani*	520	100.00	UDB0767203

Pb: Base pairs. The ITS sequence of isolate LS07 was not informative for reliable species-level identification; therefore, taxonomic assignment was based on multilocus analysis using EF1-α and BT2 markers (see Table 4).

Subsequently, a more precise taxonomic identification of fungal isolates LS5 and LS7 was undertaken. This refinement was attributable either to low sequence quality or to the limited resolving power of the ITS marker for species-level discrimination within the family Botryosphaeriaceae. Therefore, species identification was based on multilocus markers with higher discriminatory capacity, avoiding overinterpretation of ITS-based results. A molecular approach was applied using sequence data from the ITS region, elongation factor 1-alpha (EF1-α), and beta-tubulin (BT2) genes. Phylogenetic analyses and significant sequence alignments were then performed using BLAST, allowing comparison of the obtained sequences with reference records deposited in international databases (Table 4; Supplementary Materials S7–S10).

**Table 4:** Molecular identification of species corresponding to isolates LS5 and LS7 not resolved in the initial analysis.

Code	Isolate	Marker	Identified species	Sequence lenth (pb)	Identity (%)	GenBank accession
PX207462	LS05	BT2	*Lasiodiplodia theobromae*	773	98.17	PQ479076.1
PX207463	LS05	EF1-α	*Lasiodiplodia theobromae*	1415	99.87	MN461169.1
PX207464	LS07	BT2	*Lasiodiplodia theobromae*	797	100	ON373967.1
PX207465	LS07	EF1-α	*Lasiodiplodia theobromae*	1679	100	XM_035519539.1

### In vitro efficacy of antagonistic endophytic microorganisms against the etiological agent of dieback

#### Isolation of microorganisms

**Fig. 5:**
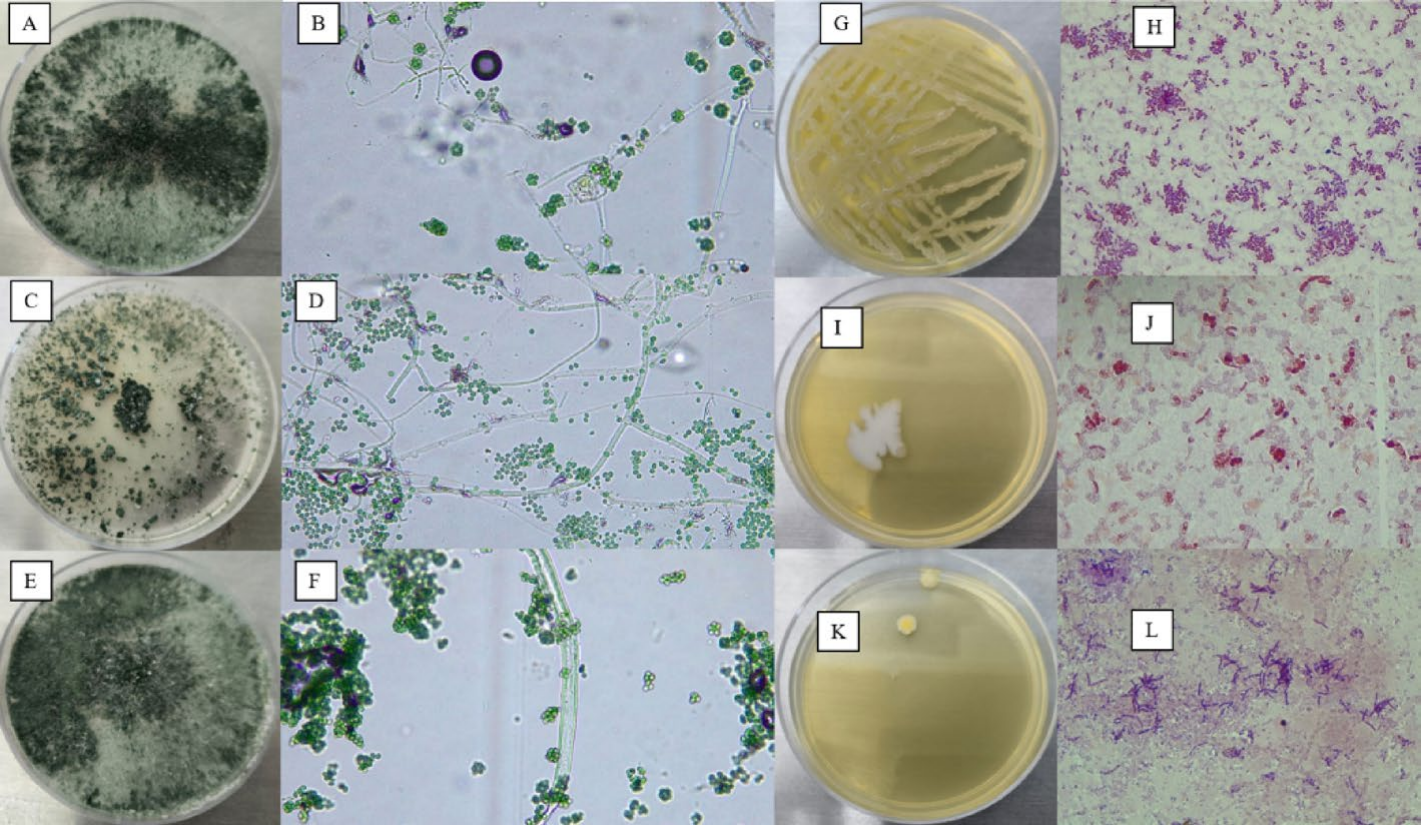
Selected *Trichoderma* and *Bacillus* isolates for the dual culture assay *Note:* (**A**) Petri dish showing the beneficial *Trichoderma* isolate Tr.01; (**B**) microscopic view of Tr.01; (**C**) Petri dish showing the beneficial *Trichoderma* isolate Tr.03; (**D**) microscopic view of Tr.03; (**E**) Petri dish showing the beneficial *Trichoderma* isolate Tr.06; (**F**) microscopic view of Tr.06. (**G**) Petri dish showing the beneficial *Bacillus* isolate BV01; (**H**) microscopic view of BV01; (**I**) Petri dish showing the beneficial *Bacillus* isolate BV02; (**J**) microscopic view of BV02; (**K**) Petri dish showing the beneficial *Bacillus* isolate BV03; (**L**) microscopic view of BV03.

*Trichoderma* was detected in 35% of the analyzed samples, from which eleven initial isolates were obtained. Following purification and preliminary screening, three strains exhibiting high colonization ability and competitive behavior against pathogenic fungi were selected, indicating potential antagonistic activity. In parallel, two bacterial cultures belonging to the genus *Bacillus* were isolated, along with an additional bacterial isolate obtained from foliar tissue, resulting in a total of three bacterial strains used in the dual confrontation assays (Fig. 5).

To further support the ecological origin and preliminary taxonomic characterization of the selected antagonistic microorganisms, detailed information on their collection site, host plant tissue, geographic coordinates, and morphological traits is presented in Tables 5 and 6. All isolates were obtained from the endosphere of healthy blueberry stems collected from commercial plantations located in the Olmos area (Lambayeque, Peru). Morphological identification was based on colony characteristics and microscopic observations following standard taxonomic keys. These data provide additional evidence of the local endophytic origin and phenotypic diversity of the evaluated biocontrol candidates.

**Table 5:** Morphological description and origin data of *Bacillus* spp. isolates obtained from the endosphere of *Vaccinium corymbosum.*

Isolate	Morphological traits					Geographic location	Site	Host	Tissue of isolation
	Gram staining	Cell shape	Motility	Colony morphology	Growth condition				
BV01	Gram-positive	Short bacilli	Motile	Irregular, whitish	Aerobic	-6.090417/-79.955427	Olmos, Lambayeque Peru	*Vaccinium corymbosum*	Stem (endosphere)
BV02	Gram-positive	Short bacilli	Motile	Irregular, whitish	Aerobic	-6.094690/-79.958105	Olmos, Lambayeque Peru	*Vaccinium corymbosum*	Stem (endosphere)
BV03	Gram-positive	Long bacilli	Motile	Round, shiny	Aerobic	-6.101822/-79.959309	Olmos, Lambayeque Peru	*Vaccinium corymbosum*	Stem (endosphere)

**Table 6:** Morphological description and origin data of *Trichoderma* spp. isolates obtained from the endosphere of *Vaccinium corymbosum.*

Isolate	Morphological traits				Geographic location	Site	Host	Tissue of isolation
	Texture	Pigmentation	Concentric rings	Conidios				
Tr.01	Compact, cottony	Dark green	Poorly defined	Green, rounded conidia 3.2—4.2 µm	− 6.076407/− 79.943834	Olmos, Lambayeque Peru	*Vaccinium corymbosum*	Stem (endosphere)
Tr.03	Slightly cottony	Dark green	Poorly defined	Green, rounded conidia 2.8—3.8 µm	− 6.071602/− 79.939853	Olmos, Lambayeque Peru	*Vaccinium corymbosum*	Stem (endosphere)
Tr.06	Compact, cottony	Dark green	Poorly defined	Green, rounded conidia 3.5—4.0 µm	− 6.071310/− 79.936978	Olmos, Lambayeque Peru	*Vaccinium corymbosum*	Stem (endosphere)

#### Antagonism assay of native Trichoderma and Bacillus strains against the causal agent of dieback during 3 and 7 DAI

**Fig. 6:**
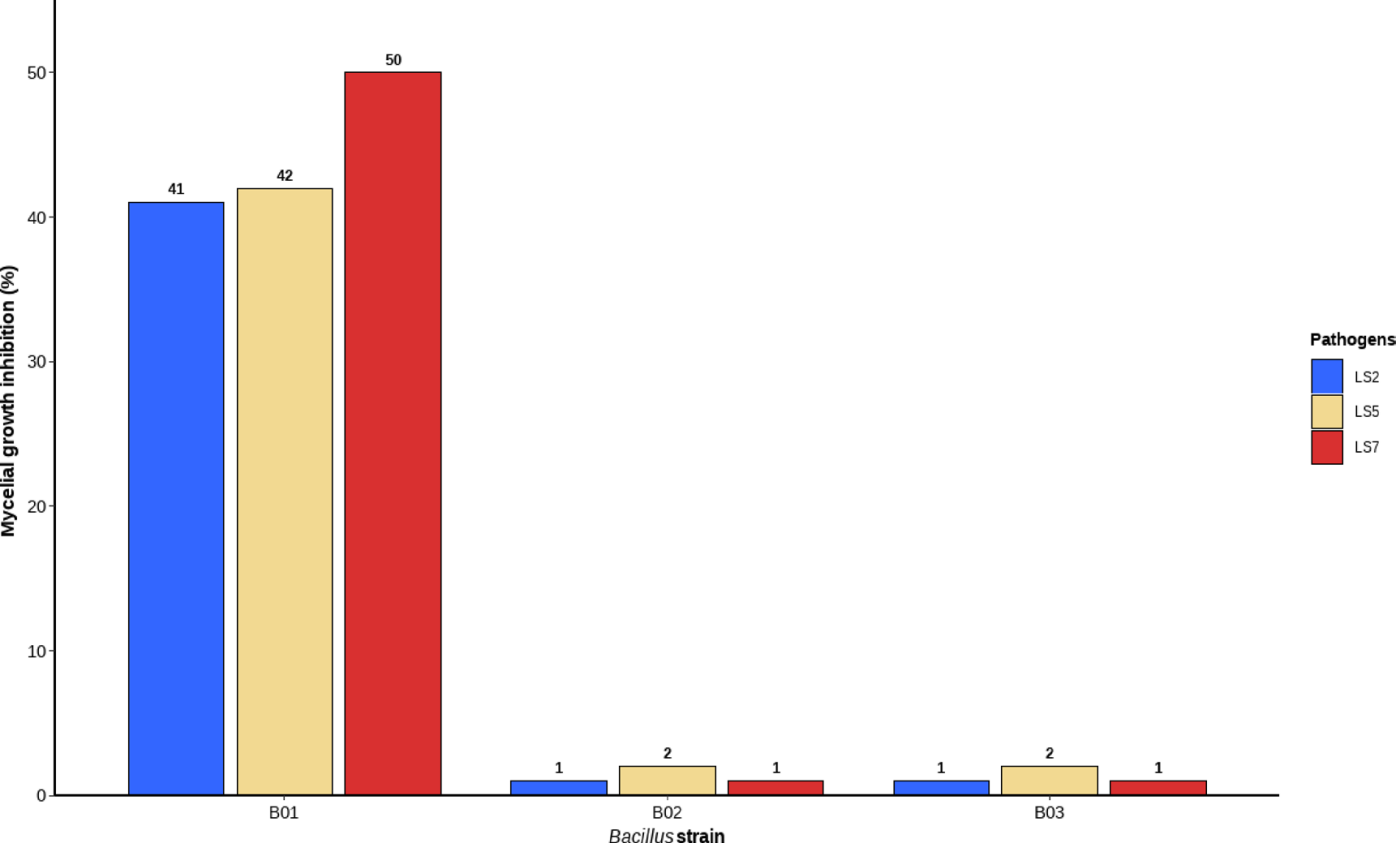
Percentage of mycelial growth inhibition of three pathogens confronted with three *Bacillus isolates* from blueberry at day 3 after inoculation.

The antagonistic activity of three *Bacillus* isolates (B01, B02, and B03) against *Lasiodiplodia* isolates LS5, LS7, and LS2 was evaluated over a three-day period using the percentage of mycelial growth inhibition (PIMG) as an indicator of biocontrol efficacy (Fig. 6). Each treatment was compared with an untreated control, allowing a direct quantification of the inhibitory effect of each bacterial isolate on fungal development. For LS2 (*L. citricola*), inhibition levels were markedly lower than those observed for LS5 and LS7. On day 1, isolates B01, B02, and B03 reached maximum inhibition values of 37%, 25%, and 18%, respectively; however, inhibition decreased on subsequent days, particularly for B02 and B03, with values falling below 16%, indicating a limited and short-lived antagonistic response against this isolate. LS5 (*L. theobromae*) also showed a measurable response, especially when confronted with B03, which achieved 51% inhibition on day 1, followed by B02 and B01 with 40% and 35%, respectively. Nevertheless, inhibitory effects declined progressively over time, with a decreasing trend observed mainly for B02 and B03. In contrast, LS7 (*L. theobromae*) exhibited the highest sensitivity to bacterial treatments, with initial inhibition values of 56%, 45%, and 46% for B01, B02, and B03, respectively. Notably, B01 maintained relatively high inhibition levels on days 2 and 3 (44% and 50%), indicating a sustained antagonistic effect against this isolate.

Isolate B01 exhibited the most consistent antagonistic activity, particularly against LS7, whereas LS2 was the least susceptible isolate. These results demonstrate differential sensitivity among Lasiodiplodia isolates and highlight the strain-dependent nature of antagonistic activity in *Bacillus*. The observed responses indicate variability in bacterial–pathogen interactions influencing mycelial growth inhibition under in vitro conditions.

**Fig. 7:**
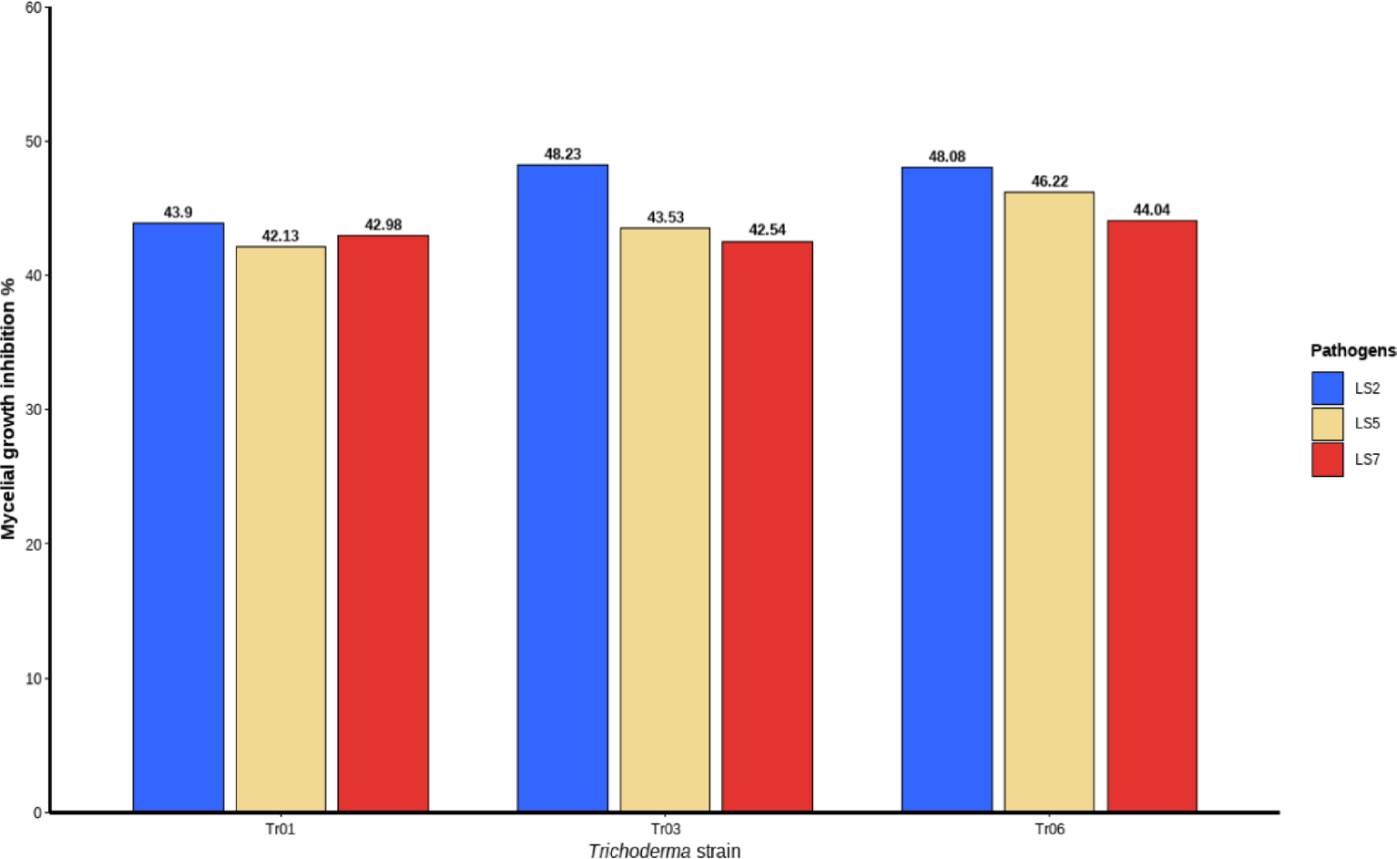
Percentage of mycelial growth inhibition of three pathogens confronted with three *Trichoderma* isolates from blueberry at day 7 after inoculation.

In dual confrontation assays, the antagonistic activity of three *Trichoderma* isolates (Tr.01, Tr.03, and Tr.06) was evaluated against *Lasiodiplodia* isolates LS2, LS5, and LS7 using the percentage of mycelial growth inhibition (PIMG) over a seven-day period (Fig. 7). Isolate LS5 fully colonized the Petri dish by day 5, whereas LS2 and LS7 reached full coverage by day 6. In all treatments, mycelial contact occurred approximately on day 2, marking the onset of direct competition. For LS2 (*L. citricola*), inhibitory response was moderate, with Tr.06 and Tr.03 showing the highest PIMG values (50.7% and 49.2%, respectively), while Tr.01 exhibited slightly lower inhibition (46.6%). In the case of LS5 (*L. theobromae*), higher mean PIMG values were recorded across treatments, with Tr.06 reaching up to 49.81%, followed by Tr.03 (49.54%) and Tr.01 (49.51%), indicating a consistent reduction in mycelial growth compared with the control. For LS7 (*L. theobromae*), Tr.06 showed the greatest inhibitory effect, with PIMG values exceeding 51% on days 3 and 4, whereas Tr.01 and Tr.03 maintained inhibition levels above 42% during the final days of the assay.

The degree of parasitism was assessed according to the scales proposed by Bell et al.^23^ and Ezziyyani et al.^27^. Isolate Tr.01 reached parasitism grade 3 when confronted with LS5, corresponding to complete overgrowth of the pathogen colony. In contrast, against LS7 and LS2, Tr.01 exhibited parasitism grade 2, indicating partial but sustained invasion. Meanwhile, Tr.03 and Tr.06 showed parasitism grade 1 against all three Lasiodiplodia isolates, which corresponds to limited invasion (approximately one-quarter of the colony surface), reflecting a lower degree of mycoparasitic interaction. These observations indicate that Tr.01 exhibited the highest level of invasive interaction, particularly against LS5, in agreement with the higher mycelial growth inhibition values recorded in the antagonism assays.

### In vitro inhibitory activity of beneficial microorganisms against Lasiodiplodia spp 4 and 7 DAI

For isolate LS2 (*L. citricola*), treatment T5 (Bio-splent®) showed the highest efficacy, maintaining inhibition levels above 96% throughout the entire evaluation period. This was followed by T4 (T-34®) and T1 (Tr.06), which consistently exhibited inhibition values above 94%. Treatment T2 (Tr.01) displayed a progressive increase in inhibition, reaching 100% during the final three days of the assay. Treatment T3 (BV01) also showed effective inhibition, although slightly lower during the final evaluations compared with T2 and T5. For isolate LS5 (*L. theobromae*), treatments T5 and T1 maintained high and stable inhibition levels, exceeding 99% PIMG from day 3 onward. Similar to the response observed against LS2, treatment T2 reached 100% inhibition during the final evaluations, confirming its high inhibitory performance. Treatment T3 exhibited a notable biocontrol effect, although with greater variability toward the end of the evaluation period. For isolate LS7 (*L. theobromae*), treatments T5 and T1 maintained stable inhibition levels above 98% from day 3 onward, reaching 100% PIMG from day 5. Treatment T2 showed a pattern comparable to that observed for the other isolates, likewise achieving 100% inhibition toward the end of the assay. In contrast, T3 presented the lowest inhibition values among treatments, although inhibition levels remained above 88% throughout all evaluations. The negative control (T6) showed no inhibition in any of the assays, confirming the validity of the experimental control (Table 7).

**Table 7:** Percentage of mycelial growth inhibition of *Lasiodiplodia* (LS2, LS5, and LS7) as affected by microorganism-based treatments, 4 and 7 DAI.

TREAT	4 DAI			7 DAI		
	LS2	LS5	LS7	LS2	LS5	LS7
T1(Tr06)	97.12 Ab	99.63 a	98.99 A	97.10 A	100.00 A	100.00 a
T2(Tr01)	99.10 A	98.28 a	98.86 A	100.00 A	100.00 A	100.00 a
T3(BV01)	94.23 B	93.93 b	92.35 C	91.22 B	90.25 B	88.18 c
T4(T-34®)	95.23 Ab	95.23 b	94.14 B	92.86 B	91.57 B	89.68 b
T5(Bio-splent®)	99.21 A	99.51 a	99.29 A	99.21 A	99.51 A	99.29 a
TES	0.00 C	0.00 c	0.00 d	0.00 C	0.00 C	0.00 d
*p-value*	< 0.001	< 0.001	< 0.001	< 0.001	< 0.001	< 0.001
CV (%)	2.41	1.03	0.58	2.17	1.06	0.47

DAI: Days after inoculation of the microorganisms; TREAT: Treatments; TES: Control. Values represent mean percentages of mycelial growth inhibition. Results correspond to analysis of variance (ANOVA) and grouping letters obtained from Tukey’s HSD test for days 4 and 7 after inoculation.

**Fig. 8:**
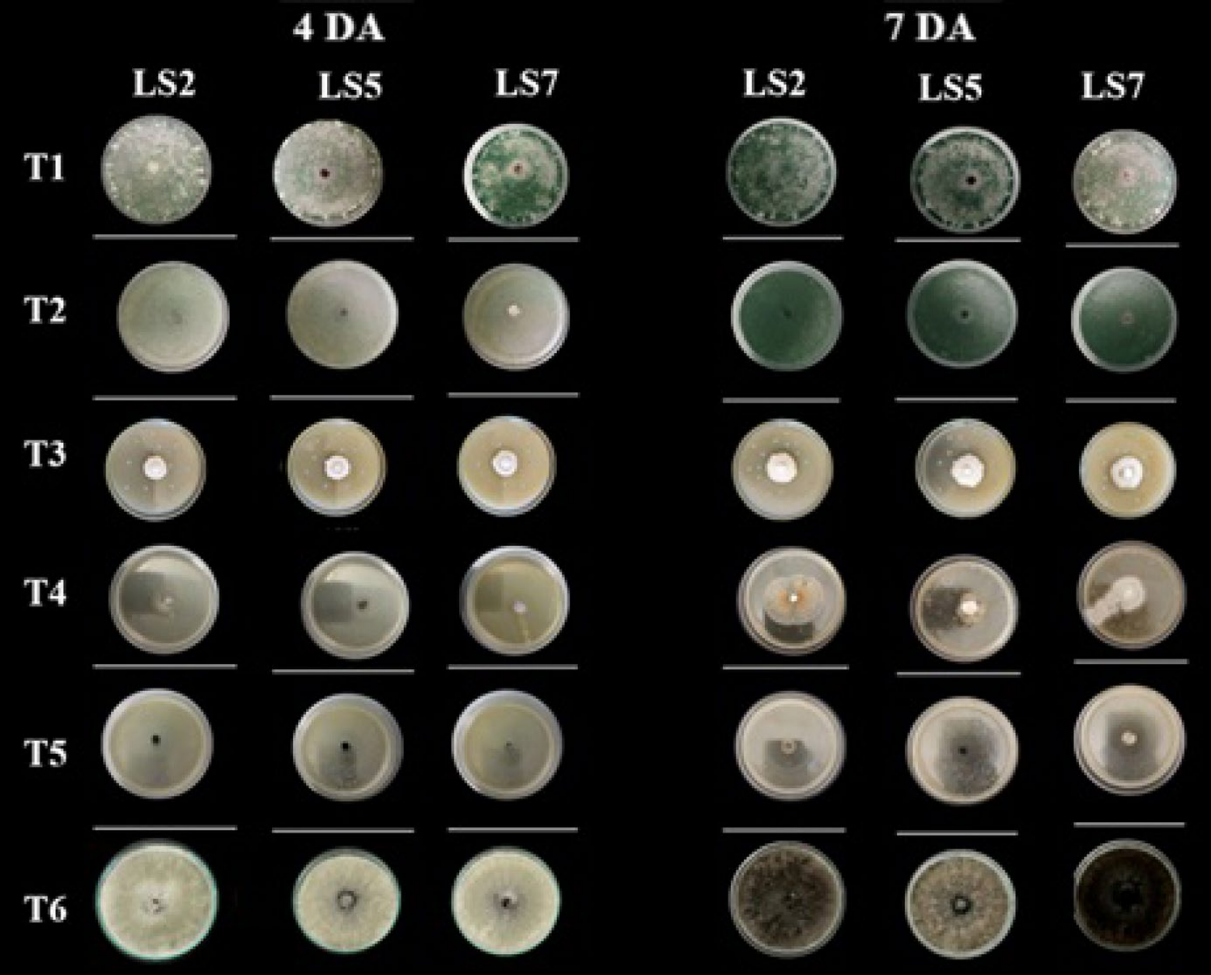
Effect of five microorganism-based treatments on the control of LS2 (*Lasiodiplodia citricola*), LS5 (*L. theobromae*), and LS7 (*L. theobromae*) using the poisoned medium assay. T6: Control. *Note: DA:* Days after inoculation of the microorganisms.

Treatments T5, T1, and T2 exhibited high and sustained mycelial growth inhibition against *Lasiodiplodia* spp. throughout the evaluation period. The stability of the inhibitory response, particularly during the intermediate and final phases of the assay, indicates a consistent inhibitory effect of these treatments under in vitro conditions (Fig. 8).

Although the evaluated microorganism-based treatments showed high and sustained inhibitory effects against Lasiodiplodia isolates, these results were obtained exclusively under controlled in vitro conditions. Therefore, they should be interpreted as an initial indication of antagonistic potential rather than direct evidence of disease suppression in planta. Further validation through greenhouse and field trials will be necessary to confirm their effectiveness under commercial blueberry production conditions.

## Discussion

### Assessment of dieback incidence in the blueberry crop

The average incidence of 7% of plants affected by dieback across the three farms evaluated in the Olmos Project indicates that this disease represents a relevant threat to the sustainability of blueberry production in northern coastal Peru. Differences among farms, with incidence values ranging from 3 to 11%, likely reflect the influence of factors related to crop age, edaphoclimatic conditions, and agronomic management practices. Although these incidence levels are lower than those reported in other major blueberry-producing countries such as Chile and China—where wood diseases may reach 15% to 45% in commercial plantations—their presence should be considered an early warning signal. This is particularly important given the chronic and cumulative nature of wood-infecting pathogens and their potential long-term impact on crop productivity and sustainability^28,29^. Furthermore, the higher susceptibility observed in the cultivars ‘Emerald’ and ‘Snowchaser’ is consistent with the well-documented genetic variability among blueberry cultivars in their responses to vascular and necrotrophic fungal pathogens. Such differences may be associated with vegetative vigor, plant architecture, and tissue regeneration capacity, factors that can influence the progression of necrosis. These findings underscore the importance of considering cultivar susceptibility as a criterion in integrated disease management programs and in the planning of new blueberry plantation.

### Isolation of the causal agent of dieback

The isolation of fungi from 100% of symptomatic samples confirms the fungal nature of the disease and indicates a high prevalence of wood-associated pathogens within the evaluated production system. The predominance of Lasiodiplodia spp. (67%) over other genera such as Neopestalotiopsis, Fusarium, and Diaporthe highlights its central role in the etiological complex of blueberry dieback. Similar findings have been reported in other studies, which identified *Pestalotiopsis* spp., *Lasiodiplodia* spp., *Neofusicoccum* spp., and Fusarium spp. as major causal agents of blueberry dieback, with a higher incidence of *Pestalotiopsis* spp^30^.

### Pathogenicity tests on blueberry plants

Pathogenicity assays conducted on blueberry stems revealed marked variation in the behavior of the evaluated fungal isolates. Isolate LS5 (*Lasiodiplodia theobromae*) exhibited the highest level of aggressiveness, showing continuous lesion progression that reached an average length of 9.99 cm at 40 days after inoculation (DAI). This was followed by LS2 (*L. citricola*, 7.39 cm) and LS7 *(L. theobromae*, 5.38 cm), indicating a strong capacity of these fungi to colonize and necrotize host tissues. The quantitative assessment of disease development using the area under the disease progress curve (AUDPC) further supported the differential aggressiveness observed among fungal isolates. The significantly higher AUDPC values recorded for isolate LS5 confirm its greater epidemiological fitness and capacity to induce rapid vascular colonization in blueberry stems. Intermediate AUDPC responses observed for LS2 and LS4 suggest moderate pathogenic potential, whereas isolates such as C3, P3 and the non-inoculated control exhibited minimal disease progress over time. These findings highlight the relevance of integrating temporal disease metrics to accurately characterize pathogenic variability within the Botryosphaeriaceae complex affecting woody crops. The pathogenic responses observed in this study are consistent with previous reports in woody crops. Úrbez-Torres et al.^31^ demonstrated that species of the genus Lasiodiplodia, particularly *L. theobromae*, can induce extensive necrosis in grapevines and other fruit crops, with rapid disease progression under conditions of elevated temperature and humidity. This description closely matches the aggressive behavior recorded for isolates LS5 (*L. theobromae*) and LS2 (*L. citricola*) in blueberry. Several studies have shown that the expression of aggressiveness in trunk disease–associated fungi is particularly pronounced in juvenile tissues. Pathogenicity trials in grapevine and other fruit trees have reported that green shoots and actively growing apices are highly susceptible, developing more extensive necrotic lesions than mature wood^31,32^. Consistently, Rodríguez-Gálvez et al.^7^ and Tennakoon et al.^33^ reported that young tissues tend to be more susceptible than mature stems, which aligns with the greater disease severity observed in young blueberry stems in the present study. In contrast, negative controls and other isolates such as C1 (Neopestalotiopsis sp.), C2 (Diaporthe sp.), C3 (Fusarium solani), and P3 (Neopestalotiopsis vaccinii) showed limited symptom development, with lesion lengths remaining below 2.5 cm throughout the entire evaluation period. This pattern suggests either non-pathogenic behavior or low virulence. These observations are consistent with the conclusions of Travadon et al.^34^, who emphasized that pathogenicity should be assessed not only based on the presence of symptoms, but also on their severity and progression over time.

### Characterization of the etiological agent of dieback

Mycelial growth of *Lasiodiplodia* isolates on PDA exhibited a strong dependence on both temperature and culture medium. Higher expansion rates observed between 25 and 30 °C are characteristics of this genus, and contribute to its predominance in warm and semi-arid regions. Alam et al.^35^, reported that *Lasiodiplodia* species exhibit increased growth within this interval, with a marked reduction under more extreme thermal conditions. Chukunda and Onyeizu^36^ and Vijay et al.^37^, additionally highlighted the ability of *L. theobromae* to grow across a wide temperature range, potentially conferring ecological advantages over other competing pathogens. At 20 °C, mycelial development was limited, whereas at 35 °C thermal stress effects were evident, although some isolates, such as LS7 and LS6, maintained considerable growth. Alam et al.^35^ reported similar behavior in studies on wood-infecting fungi associated with fruit trees (banana). In this situation, extreme temperatures significantly delayed substrate colonization, and no growth was observed at 10 or 45 °C. In addition, Chen et al.^38^ and Xu et al.^39^ demonstrated that thermal stress in *Lasiodiplodia* species induces reddish pigmentation and reduces mycelial expansion rates, negatively affecting cellular viability and sporulation. The interpretation of a stress-related response to elevated temperature is supported by the observation of reddish pigmentation in isolates LS2 and LS3 in the present study. Growth patterns on CMA differed markedly from those observed on PDA. At 25 and 30 °C, all isolates achieved complete plate colonization by day 3. Even at 20 °C, where colonization on PDA required up to 14 days, all seven isolates reached full coverage on CMA within 6 days. These findings indicate that mycelial growth is enhanced as nutrient availability in the substrate increase Chukunda and Onyeizu^36^ and Saha et al.^40^). They also observed greater development of *Lasiodiplodia* isolates on nutrient-enriched media compared to PDA in African mahogany and young tea plants. The differential behavior observed among isolates may also be related to their geographic origin or host source, suggesting the presence of locally adapted populations suited to the hot and arid conditions of the Olmos Valley. These results highlight the importance of evaluating strain-specific virulence. Therefore, biological control strategies or genetic resistance programs should be implemented to improve durability of disease management approaches.

### Antagonism assay of endophytic Trichoderma and Bacillus isolates strains against the causal agent of dieback

*Trichoderma* strains exhibited variable antagonistic responses against *Lasiodiplodia*, with Tr.01 standing out due to its pronounced mycoparasitic activity, while Tr.06 showed the highest level of mycelial growth inhibition. The inhibitory capacity of *Trichoderma* may be associated with the synthesis of hydrolytic enzymes and antifungal metabolites, mechanisms that have been extensively documented in the literature^41^. Regarding LS2 (*L. citricola*), the antagonistic microorganisms exhibited only low to moderate inhibition levels (grades 1–2). This variable response may be attributed to physiological differences among *Lasiodiplodia* strains, particularly related to the production of phenolic and inhibitory compounds that could limit the antagonistic effectiveness of *Trichoderma*^42,43^. LS7 (*L. theobromae*) exhibited an intermediate response, with high inhibition levels particularly during the initial days of interaction, suggesting an early antagonistic effect on pathogen growth. Previous studies involving *Trichoderma* spp. have reported strong suppression of *L. theobromae* during the first 3–4 days of in vitro interaction^44,45^. These findings support the notion that the colonization rate of Trichoderma represents a crucial factor in its inhibitory mechanism Confrontation assays between *Bacillus* colonies (BV01, BV02, and BV03) and *Lasiodiplodia* isolates revealed marked differences in mycelial growth inhibition (MGI). Consequently, this pathogen–antagonist intraspecific interaction significantly influences biocontrol efficacy. Additionally, such variability may be explained by genetic and metabolic differences among fungal and bacterial isolates^46^. LS7 (*L. theobromae*) showed greater susceptibility to *Bacillus* colonies, particularly on the first day of evaluation, with inhibition values of 56%, 45%, and 46% for BV01, BV02, and BV03, respectively. This pattern suggests that the bacterial antagonistic effect was more pronounced during the early stages of pathogen development, likely associated with the early synthesis of antimicrobial compounds such as surfactins, iturins, and fengycins, which are widely recognized for their antifungal activity^47^. In contrast, LS5 (*L. theobromae*) exhibited a moderate response, especially on the first day, with inhibition values of 51% for BV01 and 40% and 35% for BV02 and BV03, respectively. However, from the second day onward, a decline in inhibition was observed across all treatments. This reduction may be attributed to multiple factors related to both the antagonist and the pathogen. A decrease in the production, release, or stability of antifungal metabolites could reduce the active fraction available in the medium, directly affecting the suppressive capacity of the biocontrol agent^48^. Furthermore, several studies suggest that under continuous or recurrent exposure, pathogens may develop physiological or adaptive responses to biological pressure exerted by antagonistic microorganisms, resulting in reduced sensitivity or partial tolerance to inhibitory compounds^49^. LS2 (*L. citricola*) was the least affected isolate, showing minimal inhibition rates on the first day—37% (BV01), 25% (BV02), and 18% (BV03)—which further decreased on subsequent days. The lower effectiveness against this isolate may be associated with fungal defense mechanisms, such as the production of degradative enzymes or compounds capable of counteracting *Bacillus*-mediated antagonism^50^. The variability observed in inhibition between the antagonist and the pathogen may be influenced by the fungal strain, the antagonistic agent, and the aggressiveness and pathogenicity of the phytopathogenic fungus^51^. This suggests that inhibitory processes are multifactorial in nature and that their assessment should incorporate species-specific variables.

### In vitro inhibitory activity of beneficial microorganisms against Lasiodiplodia spp.

Treatment T5 (Bio-splent®), whose active ingredient is *Bacillus subtilis*, was the most effective throughout the seven-day evaluation period, maintaining inhibition percentages above 96%. Previous studies have highlighted the ability of *B. subtilis* to produce antifungal metabolites—such as fengycins, surfactins, and iturins—capable of degrading the cell walls of phytopathogenic fungi^47,52^. Similarly, treatments T4 (T-34®) and T1 (Tr.06), corresponding to *Trichoderma asperellum* and *Trichoderma* spp., respectively, exhibited high levels of mycelial growth inhibition in most assays, exceeding 94%. These processes were possible through multiple mechanisms, including competition for space and nutrients, production of lytic enzymes (such as chitinases and glucanases), direct mycoparasitism, and the release of antagonistic secondary metabolites and volatile compounds described by Guzmán-Guzmán et al.^53^ for *T. asperellum.* Likewise, treatment T2 (Tr.01) showed a progressive increase in efficacy, ultimately achieving complete inhibition in the final evaluations. Harman et al.^54^, also describe *Trichoderma* spp.’s capabilities enhancing their antagonistic performance as they establish and activate biocontrol mechanisms (production of hydrolytic enzymes and the induction of plant defense responses). This may explain the gradual increase in inhibition observed as the fungus consolidates its interaction with the pathogen and the crop environment. Treatment T3 (BV01) exhibited acceptable inhibition levels, although lower than those recorded for the other treatments. Despite maintaining inhibition percentages above 88%, its performance was more variable, suggesting reduced persistence of antagonistic effects or limited competitive ability against *Lasiodiplodia* species. As noted by Monte^55^, the effectiveness of a biocontrol agent depends not only on its antagonistic potential but also on its capacity for ecological adaptation. Notably, isolates LS5 and LS7 were slightly more responsive to the treatments than LS2, maintaining high and stable inhibition levels above 98% MGI from the third day onward. This differential response may be attributed to physiological or genetic differences among isolates of the same species, as reported by Logeshwari et al.^56^ in studies on mango. Overall, these findings provide preliminary evidence of the antagonistic potential of selected beneficial microorganisms against Lasiodiplodia spp. under controlled in vitro conditions. Such results contribute to the identification of promising candidates for future biological control strategies, which should be further validated under greenhouse and field conditions before practical implementation.

Although assays were performed in vitro, they provide a robust first screening for the selection of effective antagonists; however, further validation under greenhouse and field conditions is required to confirm their consistency, persistence, and practical applicability within integrated disease management programs.

Although the antagonistic isolates were identified based on morphological and ecological criteria commonly used in preliminary screening studies, molecular characterization was not performed in the present research. Therefore, future studies should incorporate multilocus or 16S/ITS-based identification to confirm the taxonomic status and genetic diversity of these beneficial microorganisms and to strengthen their potential application in integrated disease management programs.

**Fig. 9:**
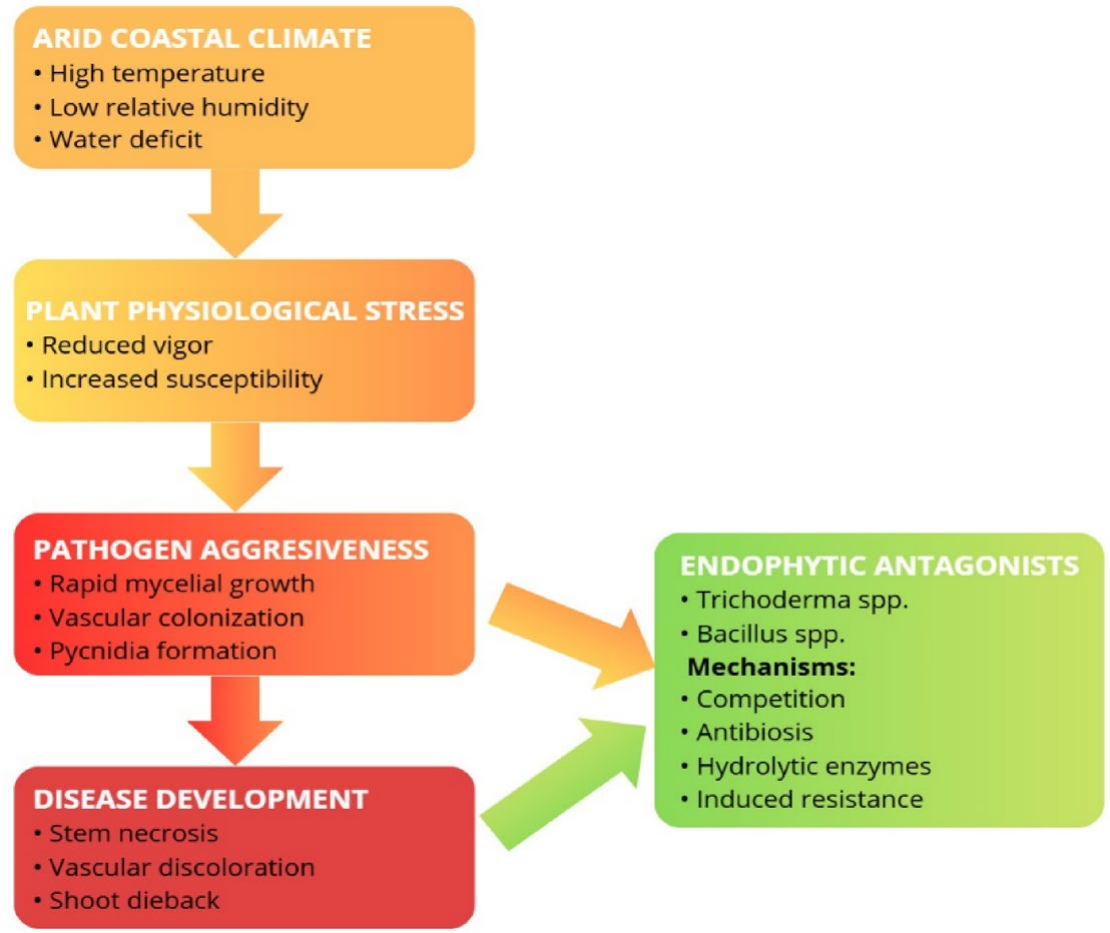
Conceptual model of environmental, pathogenic and biological interactions involved in blueberry dieback under arid coastal production conditions.

Based on the integration of pathogenicity, environmental response, and antagonistic interaction results, a conceptual interaction model is proposed (Fig. 9) to summarize the ecological mechanisms underlying dieback development and its potential biological suppression in blueberry production systems under arid coastal conditions.

This integrative framework may assist in guiding future epidemiological and biological control studies targeting trunk diseases in perennial fruit crops.

## Conclusions

Blueberry dieback represents a relevant phytosanitary constraint in the Nuevo Proyecto–Olmos area, with an average incidence of 7%, affecting both productivity and crop longevity under field conditions. The disease was primarily associated with fungi belonging to the genus *Lasiodiplodia* (67%), with *L. theobromae* standing out due to its high frequency and aggressiveness. Other genera, including *Neopestalotiopsis*, *Fusarium*, and *Diaporthe*, were detected at lower frequencies and exhibited limited symptom development, suggesting non-pathogenic behavior or low virulence under the conditions evaluated. Environmental conditions characteristic of the study area, particularly temperatures ranging between 25 and 30 °C, were associated with enhanced mycelial growth and establishment of *Lasiodiplodia* spp., which may contribute to increased disease severity in warm and semi-arid regions. Mycelial growth on CMA was faster and more homogeneous than on PDA, highlighting the influence of nutrient availability on fungal development. Endophytic microorganisms belonging to *Trichoderma* spp. and *Bacillus* spp. exhibited promising antagonistic activity under in vitro conditions, highlighting their potential as preliminary candidates for future biological control strategies. Further validation under greenhouse and field conditions will be necessary before their practical incorporation into integrated disease management programs.

## Supplementary Information

Below is the link to the electronic supplementary material.

Supplementary Material 1

## Data Availability

The obtained sequences were identified by BLAST searches against the NCBI database and deposited in GenBank under accession numbers MW299392, PP439605, PQ114133, UDB076775, PP859451, PQ126990, OQ316613, ON454616, FJ612924, and UDB07. The data supporting the findings of this study are available in the institutional repository of the National Institute of Agrarian Innovation (INIA, Peru) and can be provided upon reasonable request from the corresponding author.
