# Supplementary material for: Endophytic Trichoderma and Bacillus isolates suppress Lasiodiplodia theobromae-associated dieback in blueberry under arid coastal conditions
Source: Sci Rep. 2026 Mar 31;16:15250. doi: 10.1038/s41598-026-46484-z (PMC13181062; doi:10.1038/s41598-026-46484-z)
Supplement: Supplementary file 1 — Supplementary Material 1 [file 41598_2026_46484_MOESM1_ESM.docx]

**Supplementary Material**


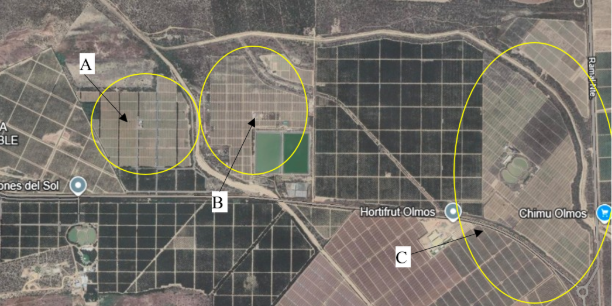


**Supplemetary material S1.** Location of the study areas: (A) B11 farm, (B) Compostera farm, and (C) Pesquera farm.

Temporal progression of dieback symptoms in blueberry plants after artificial inoculation with wood-infecting fungi under arid coastal conditions: (a) 14 days after inoculation showing initial stem necrosis; (b) 21 days showing lesion expansion and tissue browning; (c) 28 days showing advanced necrosis and partial shoot collapse; (d) 40 days showing severe vascular discoloration and complete dieback of the apical shoot.


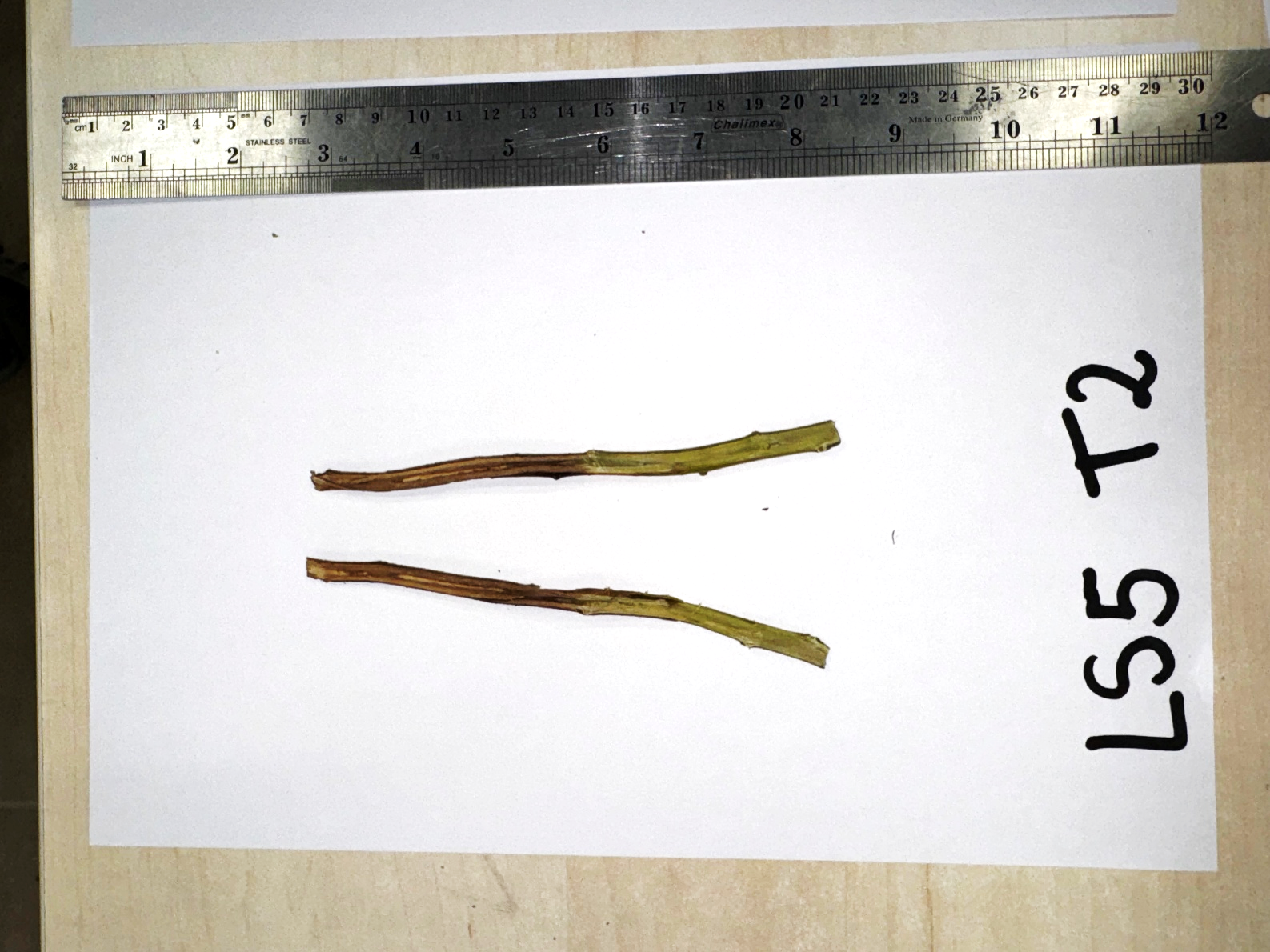

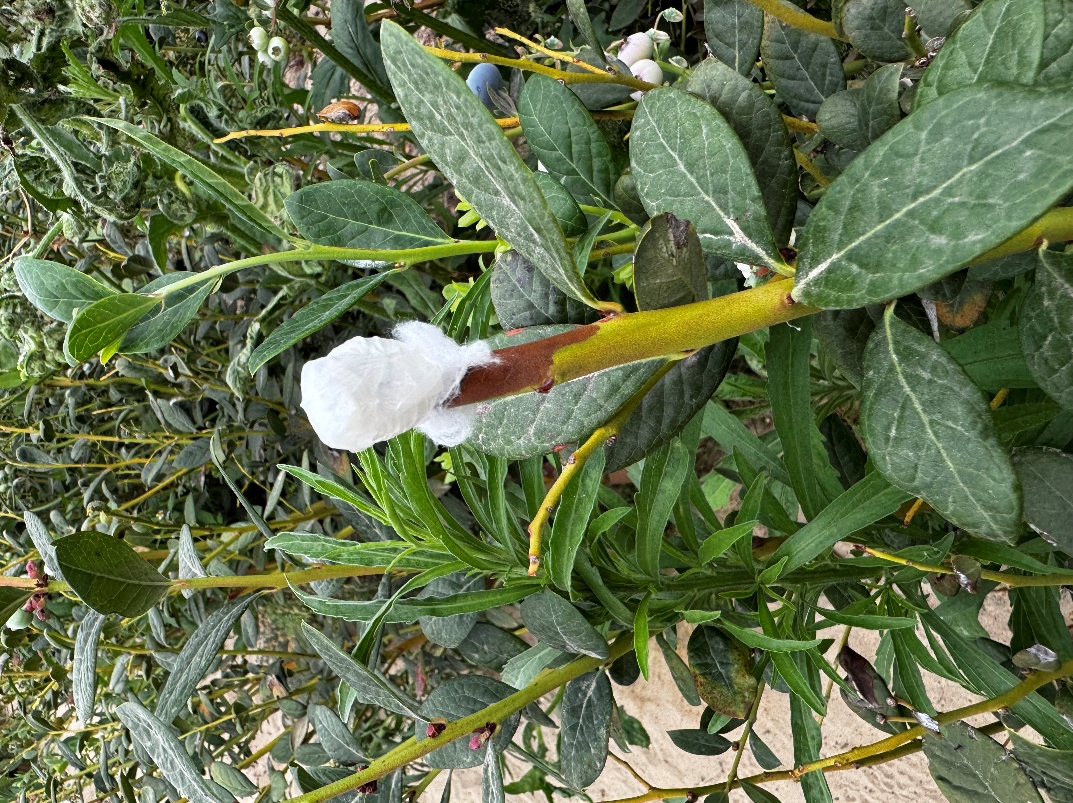


**(b)**

**(a)**

(a) Initial symptoms (b) longitudinal section


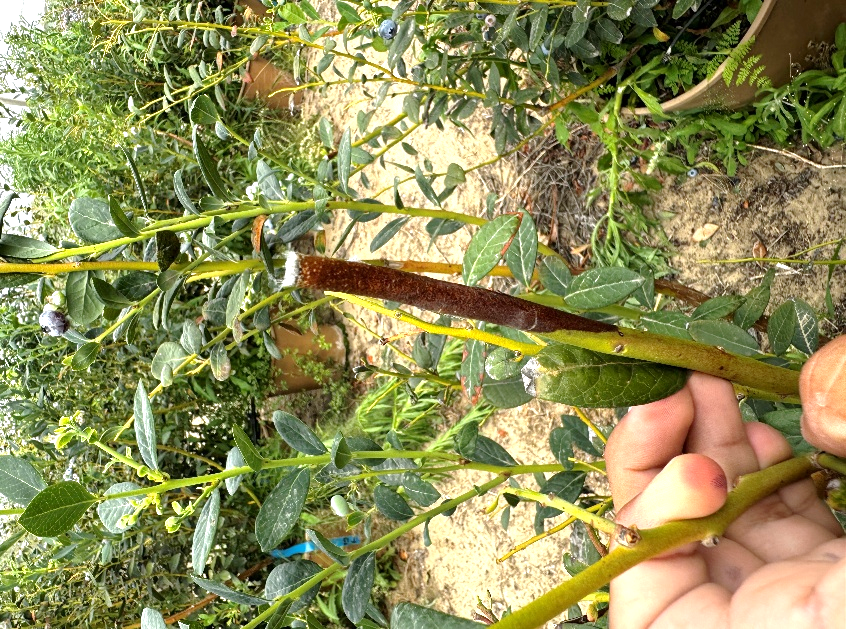

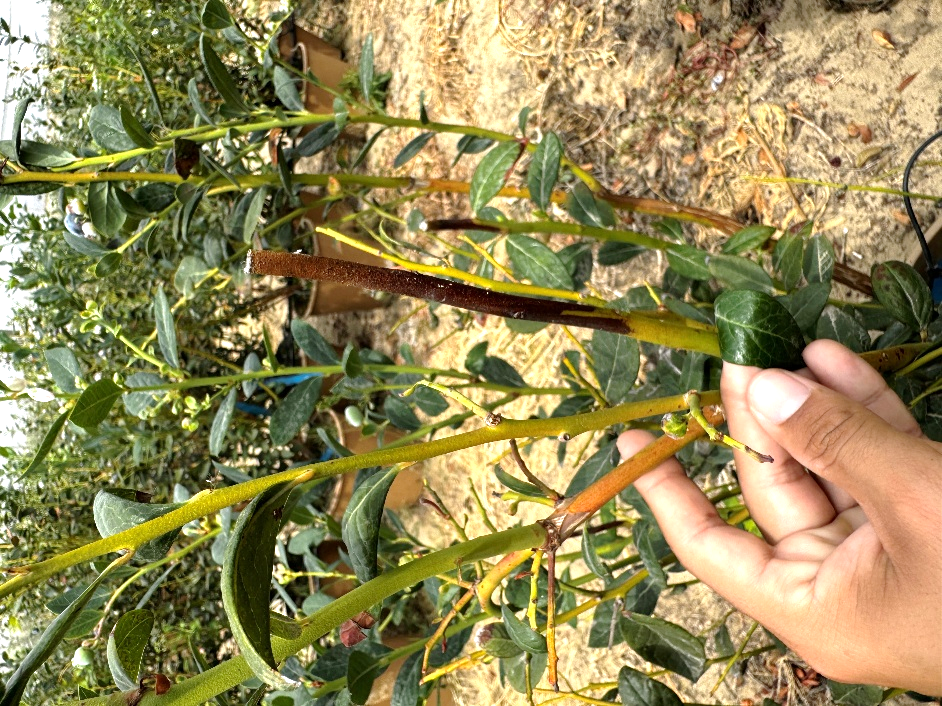


**(b)**

**(a)**

**(c)**

**(d)**


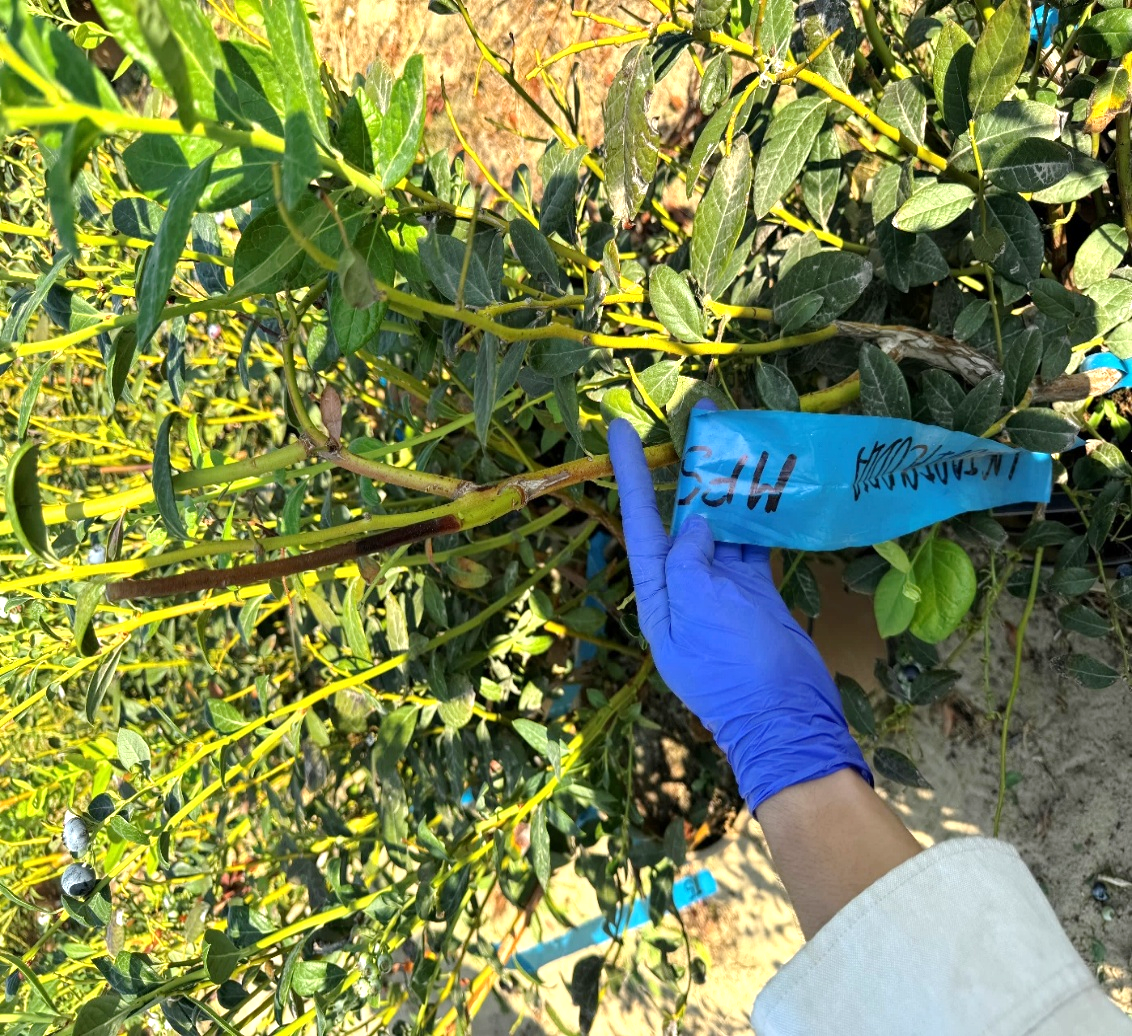

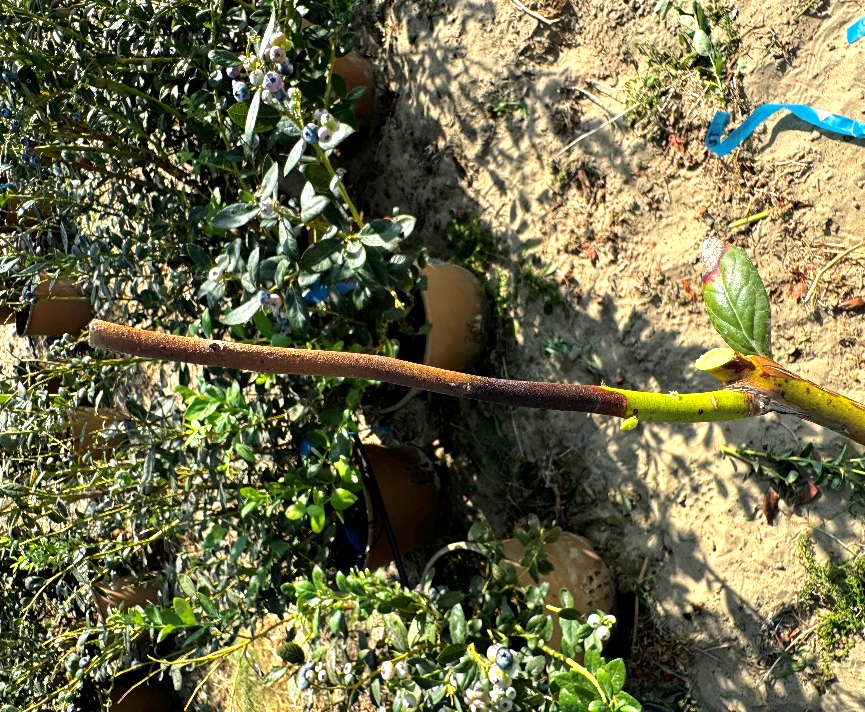


Progression of dieback disease in blueberry under arid coastal conditions: (a) 14 days, (b) 21 days, (c) 28 days, (d) 40 days.


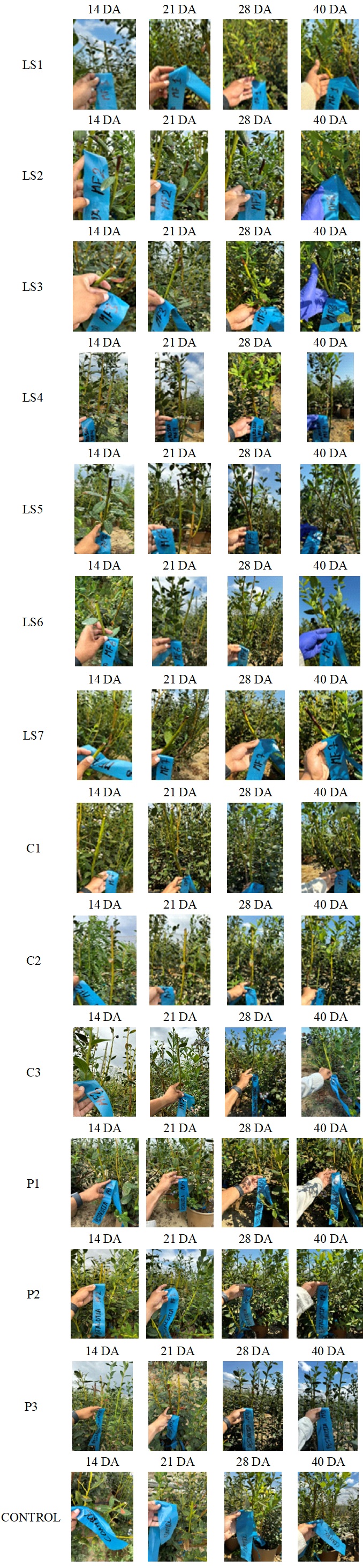

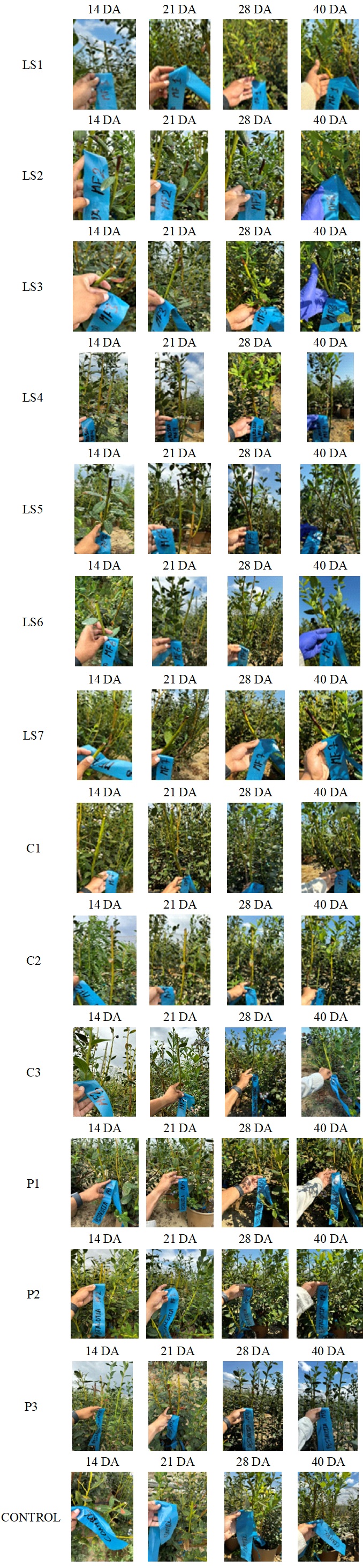


**Supplemetary material S2.** Monitoring of the pathogenicity test.

*Note*: DA = days after inoculation

| **Grade** | **Antagonistic capacity** |
| --- | --- |
| **0** | No invasion of the pathogen colony surface. |
| **1** | Invasion of one-quarter (¼) of the pathogen colony surface. |
| **2** | Invasion of one-half (½) of the pathogen colony surface |
| **3** | Complete invasion of the pathogen colony surface. |
| **4** | Complete invasion of the pathogen colony surface with sporulation over the pathogen. |

**Supplemetary material S3**. Parasitism scale according to Bell et al. (1982).


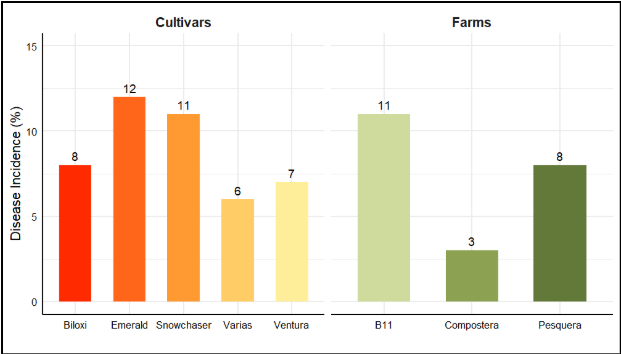


**Supplemetary material S4.** Dieback incidence (%) in blueberry cultivars and farms evaluated in the Nuevo Proyecto Olmos.

.

| TREATMENT | Evaluation days | | | | | | | | | | | |
| --- | --- | --- | --- | --- | --- | --- | --- | --- | --- | --- | --- | --- |
|  | **7 DD** | | **13 DD** | | **21 DD** | | **27 DD** | | **34 DD** | | **40 DD** | |
| CONTROL | 0,06 | h | 0,24 | f | 0,40 | e | 1,48 | d | 1,69 | d | 1,99 | fg |
| LS1 | 1,58 | bcd | 2,43 | bc | 3,25 | bc | 3,91 | c | 4,39 | c | 5,01 | bc |
| LS2 | 3,60 | a | 5,07 | a | 6,10 | a | 6,49 | ab | 6,95 | ab | 7,39 | ab |
| LS3 | 0,51 | bc | 0,84 | ef | 1,33 | d | 2,43 | cd | 2,91 | cd | 3,25 | cdefg |
| LS4 | 2,19 | b | 2,9 | b | 3,73 | b | 4,01 | bc | 4,19 | c | 4,30 | bcd |
| LS5 | 5,19 | a | 7,19 | a | 8,58 | a | 9,09 | a | 9,71 | a | 9,99 | a |
| LS6 | 1,59 | bcde | 2,26 | bcd | 3,11 | bc | 3,74 | c | 4,06 | c | 4,37 | cde |
| LS7 | 2,06 | bc | 2,83 | b | 3,60 | b | 4,01 | c | 4,78 | bc | 5,38 | bc |
| C1 | 0,78 | defg | 1,04 | de | 1,31 | d | 1,72 | d | 2,06 | d | 2,31 | defg |
| C2 | 0,96 | defg | 1,16 | cde | 1,61 | cd | 2,71 | cd | 3,29 | cd | 3,86 | cdef |
| C3 | 0,43 | g | 0,95 | ef | 1,07 | de | 1,45 | d | 1,77 | d | 2,13 | fg |
| P1 | 1,01 | cdef | 1,17 | cde | 1,27 | d | 1,72 | d | 1,97 | d | 2,12 | efg |
| P2 | 0,98 | def | 1,14 | cde | 1,23 | d | 1,45 | d | 1,67 | d | 1,84 | fg |
| P3 | 0,78 | efg | 1,08 | de | 1,31 | de | 1,58 | d | 1,63 | d | 1,66 | g |
| CV | 29,12 | ** | 30,19 | ** | 29,71 | ** | 27,45 | ** | 26,72 | ** | 28,00 | ** |

*Note*. Original data were square-root transformed prior to performing ANOVA. DD: Days after inoculation.

**Supplemetary material S5**. Evaluation of lesions on blueberry stems after pathogen inoculation


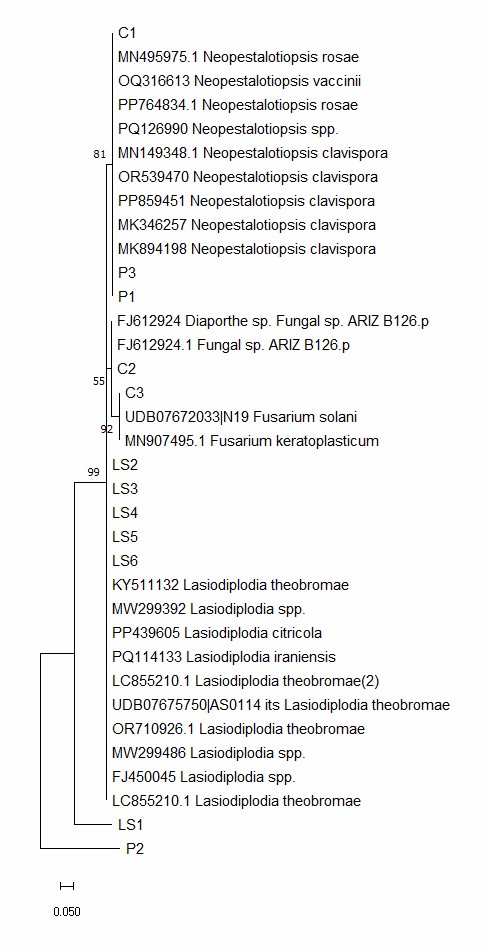


**Supplemetary material S6.** Phylogenetic tree based on ITS sequences**.**


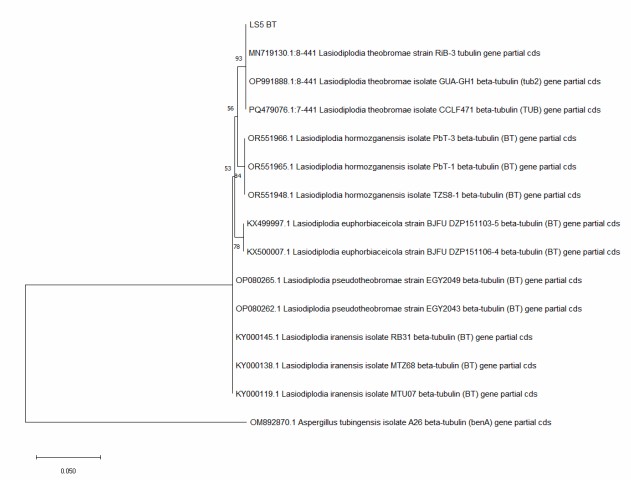


**Supplemetary material S7.** Phylogenetic tree of isolate LS5 based on the β-tubulin (BT2) gene sequence.


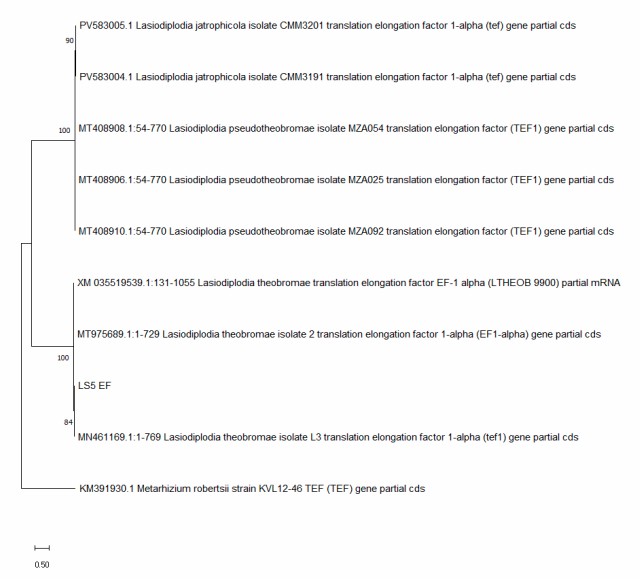


**Supplemetary material S8.** Phylogenetic tree of isolate LS5 based on the EF1-α gene sequence.


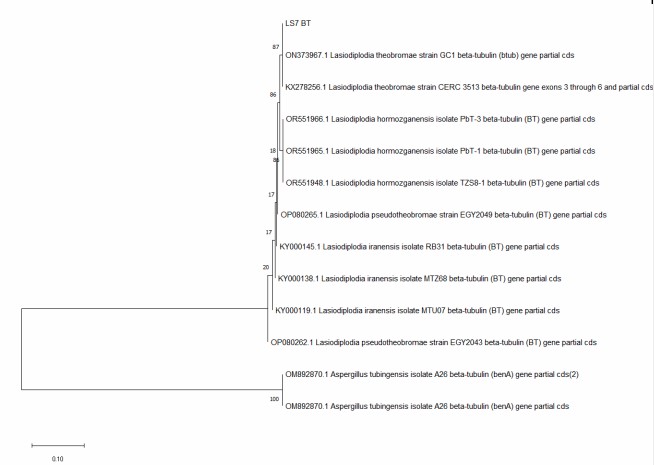


**Supplemetary material S9.** Phylogenetic tree of isolate LS7 based on the β-tubulin (BT2) gene sequence.


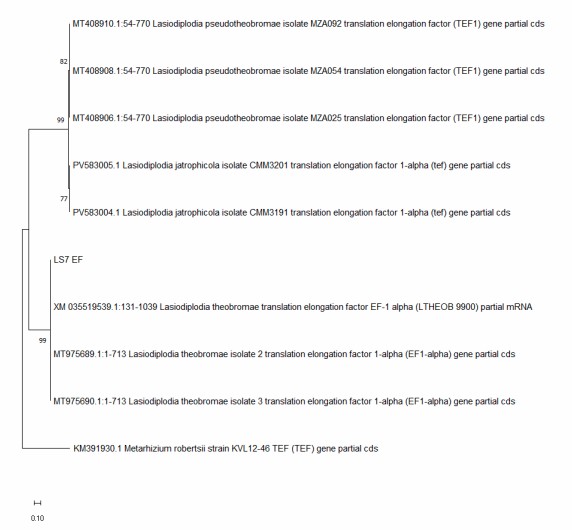


**Supplemetary material S10.** Phylogenetic tree of isolate LS7 based on the EF1-α gene sequence**.**
